# Inhibition of acetyl-CoA carboxylase impaired tubulin palmitoylation and induced spindle abnormalities

**DOI:** 10.1038/s41420-023-01301-8

**Published:** 2023-01-09

**Authors:** Chieh-Ting Fang, Hsiao-Hui Kuo, Oyundari Amartuvshin, Hwei-Jan Hsu, Sih-Long Liu, Jhong-Syuan Yao, Ling-Huei Yih

**Affiliations:** 1 Institute of Cellular and Organismic Biology, Academia Sinica, Taipei, Taiwan; 2 Molecular and Cell Biology, Taiwan International Graduate Program, Academia Sinica, Taipei, Taiwan; 3 Graduate Institute of Life Science, National Defense Medical Center, Taipei, Taiwan

**Keywords:** Mitotic spindle, Microtubules

## Abstract

Tubulin s-palmitoylation involves the thioesterification of a cysteine residue in tubulin with palmitate. The palmitate moiety is produced by the fatty acid synthesis pathway, which is rate-limited by acetyl-CoA carboxylase (ACC). While it is known that ACC is phosphorylated at serine 79 (pSer^79^) by AMPK and accumulates at the spindle pole (SP) during mitosis, a functional role for tubulin palmitoylation during mitosis has not been identified. In this study, we found that modulating pSer^79^-ACC level at the SP using AMPK agonist and inhibitor induced spindle defects. Loss of ACC function induced spindle abnormalities in cell lines and in germ cells of the *Drosophila* germarium, and palmitic acid (PA) rescued the spindle defects in the cell line treated transiently with the ACC inhibitor, TOFA. Furthermore, inhibition of protein palmitoylating or depalmitoylating enzymes also induced spindle defects. Together, these data suggested that precisely regulated cellular palmitate level and protein palmitoylation may be required for accurate spindle assembly. We then showed that tubulin was largely palmitoylated in interphase cells but less palmitoylated in mitotic cells. TOFA treatment diminished tubulin palmitoylation at doses that disrupt microtubule (MT) instability and cause spindle defects. Moreover, spindle MTs comprised of α-tubulins mutated at the reported palmitoylation site exhibited disrupted dynamic instability. We also found that TOFA enhanced the MT-targeting drug-induced spindle abnormalities and cytotoxicity. Thus, our study reveals that precise regulation of ACC during mitosis impacts tubulin palmitoylation to delicately control MT dynamic instability and spindle assembly, thereby safeguarding nuclear and cell division.

## Introduction

Microtubules (MTs), the cytoskeletal fibers composed of tubulin proteins, play essential roles in cellular division, shaping, motility and polarity. To support the diverse roles of MTs, tubulin undergoes numerous post-translational modifications (PTMs) to fine-tune MT properties and functions [1, 2]. In particular, tubulin PTMs, such as acetylation, methylation, phosphorylation, polyglutamylation, polyglycylation, and detyrosination/retyrosination, are known to modulate the stability, dynamics, mechanical properties and interaction landscape of MTs. Based on these roles, tubulin PTMs have been implicated in various MT-dependent pathophysiological processes, including ciliary functions, neuronal development, myocyte mechanical properties, mitotic/meiotic cell division, and cancer progression [1, 3–6].

S-palmitoylation (hereafter palmitoylation) is a tubulin PTM that has been studied in various cells and in vitro systems [7–11]. However, it remains largely unclear how tubulin palmitoylation affects MT properties and functions. Palmitoylation is a reversible modification that can adjust the hydrophobicity of a protein to regulate its membrane association and its activity [12, 13]. Several oncogenic proteins (e.g., RAS, EGFR, and Wnt) and tumor suppressors (e.g., SCRIB, MC1R, and Bax) also require palmitoylation to facilitate their membrane localization and execution of cellular functions [14, 15]. Besides, tubulin palmitoylation was found to be increased after androgen treatment and is required for cell proliferation in prostate cancer cells [16]. However, the roles of tubulin palmitoylation and how it is regulated during cell proliferation remain unknown.

Palmitate is the substrate used for palmitoylation and the product of successive reactions catalyzed by acetyl-CoA carboxylase (ACC) and fatty acid synthase (FASN) in the fatty acid (FA) synthesis pathway [17–19]. The two ACC isoforms in humans, cytosolic ACC1 and mitochondria-associated ACC2, catalyze the carboxylation of acetyl-CoA to produce malonyl-CoA. FASN then utilizes the malonyl-CoA produced by ACC1 together with acetyl-CoA to synthesize palmitate. Malonyl-CoA produced by ACC2 serves to allosterically inhibit carnitine palmitoyl transferase 1 (CPT1), a key enzyme for import of FAs into the mitochondria for β-oxidation. Together, the two isoforms increase the total cellular lipid level by stimulating de novo lipogenesis and inhibiting CPT1-mediated lipid oxidation. Notably, perturbed activities of ACC and FASN in yeast were shown to induce defective cytokinesis and unequal chromosome segregation during cell division [20–22]. Inhibition of ACC and FASN has also been shown to modulate cancer cell responses to MT-targeting drugs, including taxol and nocodazole [23]. In addition, ACC, the rate-limiting enzyme in FA synthesis, undergoes inhibitory phosphorylation at Ser^79^ by AMPK and localizes to the spindle pole (SP) during mitosis (from prophase through anaphase) [24, 25]. These studies imply that the FA synthesis pathway may be regulated to ensure successful mitosis progression. ACC inhibition has been demonstrated to induce FA β-oxidation [26, 27] and to elevate cellular acetyl-CoA level, thereby increasing protein acetylation [28, 29]. Yet, it remains unclear how the regulation of FA synthesis might contribute to mitotic fidelity.

In this study, we investigated whether and how FA synthesis regulates MT functions during mitosis. We showed that ACC and FA synthesis may be regulated during mitosis to control tubulin palmitoylation, MT dynamic instability, and spindle assembly. Our findings also reveal potential coordination between lipogenesis, tubulin PTMs, and MT functions that is important for mitotic cell division.

## Results

### The pSer^79^-ACC accumulates at the SP of mitotic cells

**Fig. 1:**
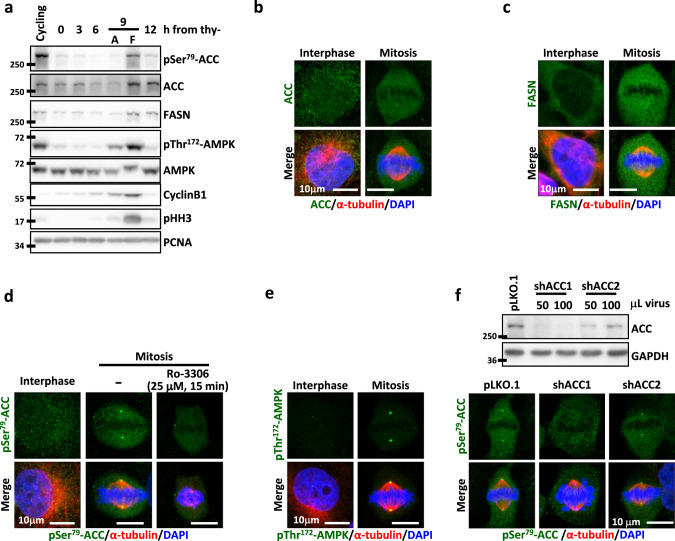
The expression and cellular distribution of ACC and FASN. ACC is phosphorylated during mitosis and localizes to the spindle pole (SP). **a** Level of the indicated proteins at each cell cycle stage. The cell cycle of CGL2 cells was enriched at each stage by double-thymidine block and release. The stages of attached G2 interphase cells (A) and floating mitotic cells (F) collected at 9 h after thymidine release (thy-) were confirmed by the expression of the G2/M markers Cyclin B1 and pHH3. **b**, **c** Subcellular localization of ACC (**b**) and FASN (**c**) in interphase and mitotic CGL2 cells. Cells were fixed and immunostained for ACC or FASN (green) as described and co-stained for α-tubulin (red) and DAPI (blue). **d** Subcellular localization of pSer^79^-ACC in interphase cells, untreated mitotic cells and mitotic cells treated with Ro-3306 as indicated. Cells were stained for pSer^79^-ACC (green), α-tubulin (red), and DAPI (blue). **e** Subcellular localization of pThr^172^-AMPK in interphase and mitotic cells. Cells were stained for pThr^172^-AMPK (green), α-tubulin (red), and DAPI (blue). **f**, top: Western blots show the efficiency of ACC depletion by shRNAs. Cells were transduced with shRNA targeting either ACC1 (shACC1) or ACC2 (shACC2) and the lysates were probed with an ACC antibody recognizing both forms. Cells transduced with the empty vector pLKO.1 were used as the control. The volumes of the shRNA-containing virions are indicated. **f**, bottom: Subcellular localization of pSer^79^-ACC in mitotic cells after transduction with control vector (pLKO.1), shACC1 or shACC2. Cells were stained for pSer^79^-ACC (green), α-tubulin (red), and DAPI (blue).

To characterize the mitotic regulation of FA synthesis pathway, we examined the protein levels and subcellular localization of ACC and FASN in CGL2 cells. The AMPK-mediated inhibitory phosphorylation of ACC at Ser^79^ (pSer^79^-ACC) [30] was also examined. Increased ACC and FASN were found in mitotic cells expressing G2/M markers Cyclin B1 and phospho-histone-H3 (pHH3) (Fig. 1a, 9 h from thy-). Significantly increased pSer^79^-ACC was also found in mitotic cells, concurrent with AMPK phosphorylation at Thr^172^ (pThr^172^-AMPK); both pSer^79^-ACC and pThr^172^-AMPK returned to baseline expression after mitosis exit when pHH3 and Cyclin B1 were reduced (Fig. 1a, 12 h from thy-). These observations are consistent with previous findings reporting the AMPK-mediated ACC phosphorylation during mitosis [24, 25].

While ACC and FASN exhibited uniform cytoplasmic localization in interphase and mitotic cells (Fig. 1b, c), both pSer^79^-ACC and pThr^172^-AMPK accumulated at the SP of mitotic cells at prophase to metaphase without showing the distinct pattern in interphase cells (Fig. 1d, e). The SP-localization of pSer^79^-ACC was abrogated by acute treatment with the CDK1 inhibitor, Ro-3306 (Fig. 1d, Ro-3306), confirming the mitosis-specific ACC phosphorylation. Thus, the role of ACC was further examined with shRNA-mediated depletion. shRNAs targeting each of the two ACC forms efficiently depleted ACC proteins (Fig. 1f, top), while SP-accumulation of pSer^79^-ACC was abrogated by depletion of ACC1 but not ACC2 (Fig. 1f, bottom), suggesting that ACC1 is the major form to be phosphorylated and localized to the SP. This mitosis-specific, SP-localized, and AMPK-mediated inhibitory phosphorylation of ACC1 implied that ACC function might be stringently regulated during mitosis to control spindle assembly.

### Disrupting ACC activity induces defects in mitotic spindles

**Fig. 2:**
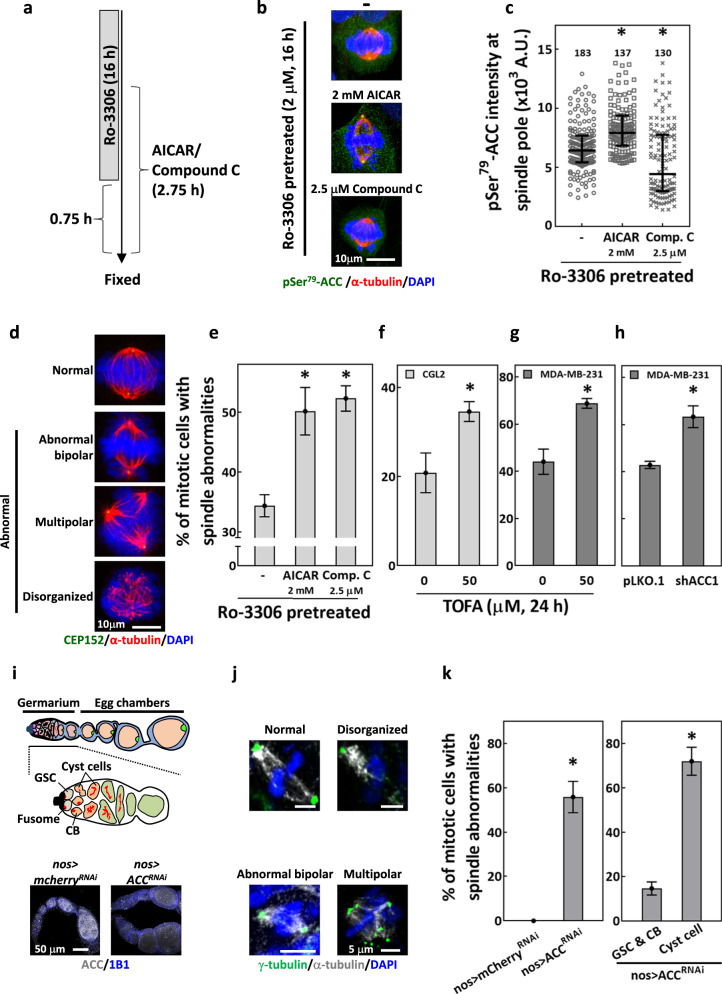
Disrupting ACC activity induces defects in mitotic spindles. **a**–**e** Disruption of ACC-Ser^79^ phosphorylation-induced spindle defects. **a** Protocol for Ro-3306 block and release and treatments of AICAR and compound C. The cell cycle of CGL2 cells was arrested and synchronized before mitosis entry by 16-h Ro-3306 treatment. For the last 2 h of Ro-3306 incubation, AICAR or compound C was added into the culture medium. After the 16-h incubation, Ro-3306 was washed away and cells were kept in medium containing AICAR or compound C for another 45 min. **b** Representative images of the Ro-3306-block-and-release-enriched mitotic cells; cells were untreated (−) or treated as indicated and stained for pSer^79^-ACC (green), α-tubulin (red), and with DAPI (blue). **c** AICAR increased pSer^79^-ACC, while compound C decreased the level of pSer^79^-ACC at the SP. The relative intensity of pSer^79^-ACC at the SP of the mitotic cells (as in **b**) was measured and presented in the scatter plot with the interquartile distribution from two independent experiments. The numbers above indicate the number of the SP measured. *P < 0.05 compared to untreated by Mann–Whitney Rank Sum test. **d** Representative images of mitotic cells with normal spindle or abnormal spindles stained for CEP152 (green), α-tubulin (red) and DAPI (blue). **e** Percentages of abnormal spindle-containing mitotic cells collected after the protocol described in (**a**) are shown as mean ± SD of at least 600 cells from two independent experiments. *P < 0.05 compared to untreated by Student’s *t* test. **f**, **g** ACC inhibition by TOFA-induced mitotic spindle abnormalities. Percentages of abnormal spindle-containing mitotic cells, untreated or treated with TOFA as indicated, are shown as mean ± SD of at least 400 cells from two independent experiments for CGL2 (**f**) and MDA-MB-231 (**g**). *P < 0.05 compared to untreated by Student’s *t* test. **h** Percentage of control (pLKO.1) and ACC1-depleted (shACC1) mitotic MDA-MB-231 cells with abnormal spindle are shown as mean ± SD of at least 400 cells from two independent experiments. *P < 0.05 compared to pLKO.1 by Student’s *t* test. **i**–**k** Knockdown of ACC-induced spindle abnormalities in *Drosophila* germaria. **i**, top: The schematic illustration of *Drosophila* germarium and egg chambers (upper scheme) and the magnified view of the germarium (lower scheme). *Drosophila* germarium each house 2–3 germline stem cells (GSCs) that asymmetrically divide to generate cystoblasts (CBs); each CB then undergoes four rounds of incomplete division to generate a 16-cell cyst. **i**, bottom: One-week-old control (*nos* > *mCherry*^*RNAi*^) and ACC knockdown (*nos* > *ACC*^*RNAi*^) ovarioles were stained for ACC (gray) and co-stained for 1B1 (blue, somatic cell membranes) to examine knockdown efficiency. **j** Representative images of the indicated types of mitotic spindles in *Drosophila* germ cells stained for γ-tubulin (green), α-tubulin (gray), and DAPI (blue). **k**, left: Percentage of mitotic cells in *nos* > *mCherry*^*RNAi*^ and *nos* > *ACC*^*RNAi*^ germ cells within germaria with abnormal spindles (including abnormal bipolar spindle, disorganized spindle, and multipolar spindle; as shown in **j**). Mean ± SD of at least 100 cells from two independent experiments is shown. **k**, right: The mitotic germ cells in *nos* > *ACC*^*RNAi*^ germaria were identified as GSCs, CBs, and Cyst cells, and the percentages of mitotic cells with abnormal spindles from each cell type are shown as mean ± SD. *P < 0.05 compared to pLKO.1 by Student’s *t* test.

To infer the roles of ACC in mitotic spindle assembly, we then modulate the activity of its upstream kinase, AMPK. As shown in Fig. 2a–c, SP-localization of pSer^79^-ACC was significantly enhanced by AICAR (AMPK agonist [31]), but decreased by compound C (AMPK inhibitor [32]). Importantly, both AICAR- and compound C-treated samples had significantly increased percentages of mitotic cells with abnormal spindles (Fig. 2d, e), suggesting that both increased and decreased level of ACC phosphorylation are correlated with the mitotic spindle defects. In addition, inhibition of ACC with TOFA [33] or depletion of ACC1 significantly increased the percentage of mitotic cells with spindle abnormalities (Fig. 2f–h). These data suggested that phosphorylation of ACC may be stringently and dynamically regulated at a certain level during mitosis to ensure proper mitotic spindle assembly.

To test whether ACC regulates mitosis at the tissue level, we examined the mitotic spindle in the *Drosophila* female germline with ACC knockdown using *nos-GAL4*. ACC was found enriched in germ cells located within the germarium and egg chambers in the ovariole of control *nos* > *mcherry*^*RNAi*^ flies but was dramatically reduced in those of *nos* > *ACC*^*RNAi*^ flies (Fig. 2i, top scheme and bottom images). We found approximately 55% of mitotic germ cells in the germarium (*nos* > *ACC*^*RNAi*^) exhibited abnormal spindles, while none was observed in control germ cells (*nos* > *mCherry*^*RNAi*^) (Fig. 2j, k, left). Further examination in each germ cell type (Fig. 2k, right) revealed that ACC knockdown caused fourfold higher abnormal spindles in cyst cells (more than 60%) than in germline stem cells (GSCs) and cystoblasts (CBs) (15%). Since CBs undergo four rounds of mitotic division to generate cyst cells, the fourfold increase in spindle defects in ACC-lacking cyst cells reflected that ACC is required for proper bipolar spindle assembly to support cell division during oogenesis in the *Drosophila* germarium. Taken together, these data suggest that precise regulation of ACC1 function is required for accurate spindle assembly during mitosis.

### Precise regulation of protein palmitoylation is required for spindle assembly

**Fig. 3:**
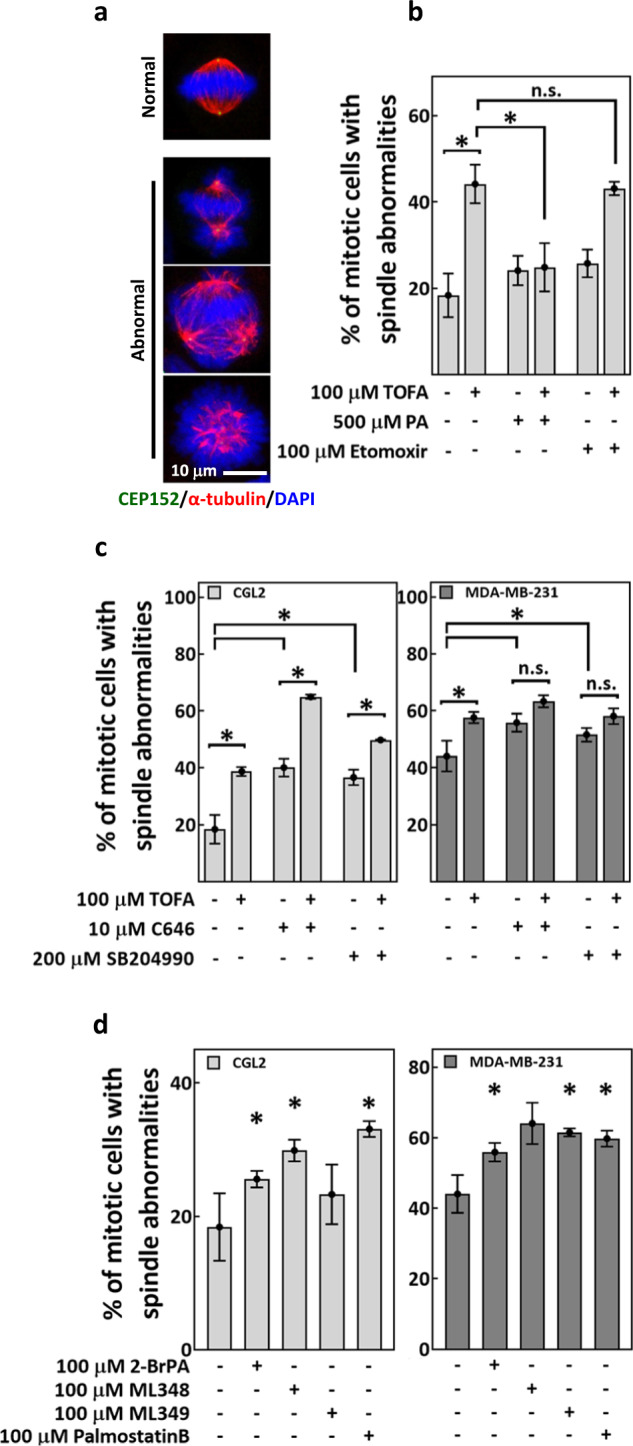
Precise regulation of protein palmitoylation is required for spindle assembly. Exogenous palmitate, but not β-oxidation inhibitor and not protein acetylation-disrupting agents, rescued TOFA-induced spindle defects. **a** Representative images of normal and abnormal mitotic spindles. Cells were stained for CEP152 (green), α-tubulin (red) and DAPI (blue). **b** CGL2 cells were treated for 1 h with TOFA, PA and etomoxir, either alone or in combination as indicated. Treated cells were then fixed and stained for spindle analysis. Percentages of mitotic cells with abnormal spindles (as in **a**) are shown as mean ± SD of at least 450 cells from three independent experiments. *P < 0.05 by Student’s *t* test; n.s. not significant. **c** CGL2 cells (left) and MDA-MB-231 cells (right) were treated for 1 h with TOFA, C646, and SB204990, either alone or in combination as indicated. Treated cells were fixed and stained for spindle analysis. Percentages of mitotic cells with abnormal spindles (as in **a**) are shown as mean ± SD of at least 600 cells from two independent experiments. *P < 0.05 by Student’s *t* test; n.s. not significant. **d** CGL2 cells (left) and MDA-MB-231 cells (right) were treated for 1 h with 2-BrPA, ML348, ML349, and palmostatin B as indicated. Treated cells were fixed and stained for spindle analysis. Percentages of cells with abnormal spindles (as in **a**) are shown as mean ± SD of at least 300 cells from two independent experiments. *P < 0.05 compared to untreated (−) by Student’s *t* test.

Since palmitate is the product of FA synthesis [19], we tested whether palmitate acts downstream of ACC in spindle assembly using combined treatment of TOFA and PA, the exogenous source of palmitate. TOFA was reported to reduce FA synthesis within 2 h [33] and we thus adopted less than 4 h treatments to monitor the immediate mitotic effects. We found that 1-h treatment of TOFA significantly increased spindle abnormalities (Fig. 3a, b). While PA alone did not increase spindle defects, its treatment in combination with TOFA significantly reduced cells containing spindle defects to the level of controls (Fig. 3b). These results implied that palmitate synthesis may play roles in ACC-dependent spindle assembly.

Malonyl-CoA, the immediate product of ACC, is an inhibitor of the CPT1-mediated FA β-oxidation [17]. We found that etomoxir, the chemical inhibitor of CPT1-mediated FA β-oxidation [34], neither induced spindle defects when treated alone nor did it rescue TOFA-induced spindle defects when treated in combination with TOFA (Fig. 3b), suggesting that the mitotic effects of ACC may be independent of CPT1-mediated β-oxidation.

Elevated acetyl-CoA level and increased protein acetylation are the potential downstream effects of ACC deficiency [28, 29, 35]. C646, the p300 acetyltransferase inhibitor [36], and SB204990, an inhibitor of ATP-citrate lyase (ACLY) that synthesizes acetyl-CoA, [37], were thus examined for their effects on TOFA-induced spindle defects. In CGL2 cells (Fig. 3c, CGL2), C646 or SB204990 in combination with TOFA additively induced spindle abnormalities compared to single treatment of each drug. In MDA-MB-231 cells (Fig. 3c, MDA-MB-231), neither C646 nor SB204990 in combination with TOFA further enhanced spindle abnormalities compared to single treatment of each drug. Thus, C646 and SB204990 exhibit no antagonizing effects on TOFA-induced spindle defects in both cell lines. Since palmitate, but not C646 and not SB204990, rescued cells from TOFA-induced spindle defects, we concluded that lack of palmitate may play a role in spindle defects induced by disruptions in FA synthesis, independent of coincident effects on protein acetylation and cellular acetyl-CoA level.

Palmitate serves as the substrate for protein palmitoylation [12, 38] that modulates protein functions. We found that 1-h treatment of 2-bromopalmitate (2-BrPA, an inhibitor of protein palmitoylation [39]) as well as ML348, ML349, and palmostatin B (protein depalmitoylation inhibitors [40, 41]) all increased abnormal spindle-containing cells both in CGL2 (Fig. 3d, CGL2) and MDA-MB-231 (Fig. 3d, MDA-MB-231). Thus, our results suggested that inhibiting either protein palmitoylation or depalmitoylation can disrupt spindle assembly. Furthermore, these data implied that precise regulation of protein palmitoylation is required for bipolar spindle assembly.

### A fraction of palmitoylated proteins colocalize with α-tubulin

**Fig. 4:**
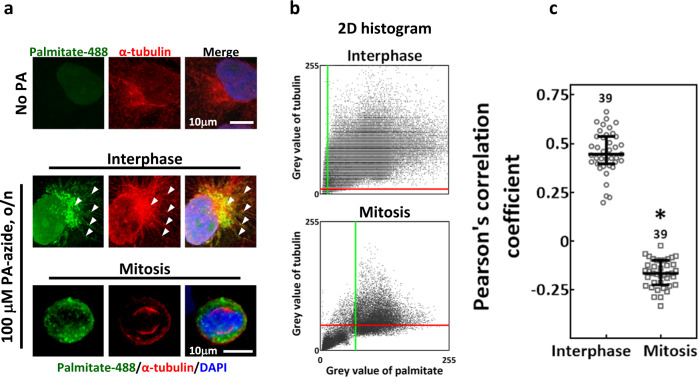
Colocalization of palmitoylated proteins with α-tubulin. CGL2 cells were incubated with PA-azide overnight and subjected to click chemistry-mediated conjugation of AlexaFluor-488 to the palmitoyl-proteins. Cells without PA-azide incubation (No PA) served as the control. **a** Representative images show control cells (No PA) and the subcellular distribution of palmitoylated proteins (palmitate-488, green) in interphase and mitotic cells that were co-stained for α-tubulin (red) and DAPI (blue). The arrowheads indicate regions of colocalization between palmitate and α-tubulin. **b** The 2D histograms show the distributions of pixel intensities for α-tubulin and of palmitate in the corresponding representative images of interphase and mitotic cells shown in (**a**). The red and green lines indicate the intensity thresholds set for tubulin and palmitate, respectively. **c** The level of palmitate-α-tubulin colocalization was assayed by Pearson’s correlation coefficient. The coefficient calculations indicate a significantly lower level of colocalization in mitosis than in interphase. The numbers above indicate the number of cells assayed from two independent experiments. *P < 0.05 by Mann–Whitney rank-sum test.

To infer the role of protein palmitoylation in mitotic spindle assembly, we labeled the palmitoylated proteins as described [42] and examined their subcellular distribution. Since tubulin plays essential roles in spindle assembly and is known to be palmitoylated [9, 11], α-tubulin was counterstained. We found that the palmitate in interphase cells exhibited both granular and fibrillar structures that were partially colocalized with α-tubulin (Fig. 4a, interphase); however, the palmitate in mitotic cells exhibited only granular structures without obvious α-tubulin colocalization (Fig. 4a, mitosis). 2D histograms of the above-threshold intensities of palmitate and tubulin revealed a clear positive correlation in interphase cells but not in mitotic cells (Fig. 4b). We found a significantly lower Pearson’s correlation coefficient in mitotic cells than that in interphase cells (Fig. 4c), suggesting reduced palmitate-α-tubulin colocalization during mitosis. These data implied that a fraction of palmitoylated proteins may be associated with MTs, but the level of association is greatly reduced after mitosis entry.

### α-tubulin is less palmitoylated in mitosis and ACC inhibition further decreases α-tubulin palmitoylation

**Fig. 5:**
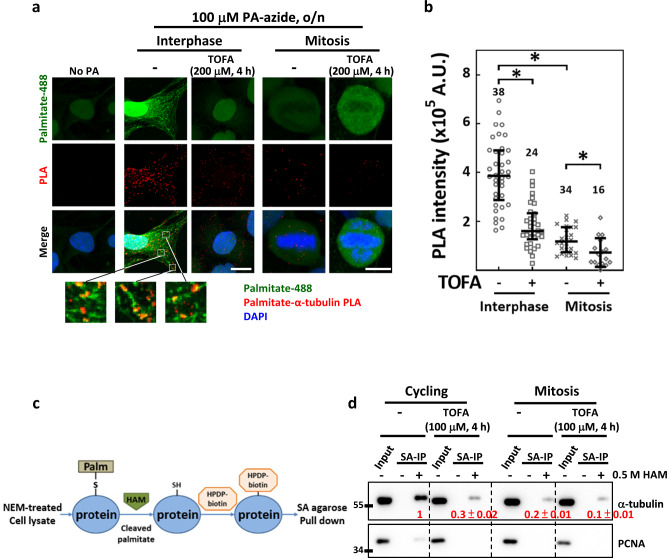
TOFA reduces tubulin palmitoylation both in interphase and mitosis. Tubulin palmitoylation was reduced during mitosis and further decreased by acute TOFA treatment. **a** The PLA between α-tubulin and click-labeled palmitate (palmitate-488) was performed using CGL2 cells. The representative images show the control cell (No PA) and the PLA signal between α-tubulin and palmitate (palmitate-α-tubulin PLA) in interphase and mitotic cells that were untreated (−) or treated with TOFA as indicated. The insets below show magnified views of regions with colocalization between PLA signals and palmitate-488. **b** Relative intensity of palmitate-α-tubulin PLA in a single cell was measured. The scatter plot shows the interquartile distribution of the PLA intensity in the group of cells described in (**a**). The numbers above indicate the numbers of cells assayed from two independent experiments. *P < 0.05 by Mann–Whitney rank-sum test. **c** Illustration of the acyl-biotin exchange assay. **d** Palmitoylated proteins in MDA-MB-231 cells were purified using acyl-biotin exchange assay and then subjected to western blotting for α-tubulin detection. HAM addition (+) is required for palmitate cleavage, biotinylation, and subsequent purification of the palmitoylated proteins, and the omission of HAM (−) served as the negative control. Input lanes show equal amounts of total tubulin in the cell lysates were applied in each reaction. The western blots show the levels of total cellular tubulin (Input) and palmitoylated fractions of tubulin purified by streptavidin agarose (SA-IP) from total cell lysates (Input) of untreated (−) and TOFA-treated cycling and enriched mitotic cells. The input tubulin was first normalized with PCNA to adjust loading variations and then served to normalize the purified tubulin. The values below each band indicate the level of purified α-tubulin after normalizing to input tubulin, relative to untreated cycling cells; mean ± SD from three independent experiments is shown.

To see whether FA synthesis affects palmitate-MT association, we quantitatively measured the level of palmitate-α-tubulin colocalization with or without TOFA treatment by proximity ligation assay (PLA) [42, 43], using No PA group as the control (Fig. 5a). Notably, the PLA signals showed colocalization with the palmitate (Fig. 5a, bottom). Four-hour TOFA treatments significantly reduced the PLA signal in interphase cells (Fig. 5a, b, interphase), suggesting disrupted palmitate-α-tubulin association. In addition, the PLA intensity in mitotic cells was significantly lower than that in interphase cells (Fig. 5a, b, mitosis) and was further decreased by 4-h TOFA treatment (Fig. 5a, b, TOFA). These data suggest that the palmitate-α-tubulin association is decreased after mitosis entry and that ACC inhibition may disrupt the palmitate-α-tubulin association in both interphase and mitotic cells.

To confirm that tubulin is palmitoylated in cells, we performed the acyl-biotin exchange assay (Fig. 5c) [44] to purify palmitoylated proteins. As shown in Fig. 5d, we found that α-tubulin was readily purified from lysates of cycling MDA-MB-231 cells, indicating that a significant fraction of α-tubulin is palmitoylated. Consistently, 4-h TOFA treatment greatly reduced the purified palmitoylated α-tubulin. In addition, the level of α-tubulin purified from mitotic cell lysates was also greatly decreased compared to untreated cycling cell lysates and was also further decreased by 4-h TOFA treatment. Quantification of the protein bands revealed the relative levels of palmitoylated tubulin in lysates of untreated cycling (set at 1), TOFA-treated cycling (0.3 ± 0.02), untreated mitotic (0.2 ± 0.01) and TOFA-treated mitotic cells (0.1 ± 0.01), consistent with the relative levels of the palmitate-α-tubulin PLA intensity in these cells (Fig. 5a, b). These data suggest that the level of tubulin palmitoylation is reduced after mitosis entry and can be further reduced by TOFA treatment. Since MTs reorganize at G2/M transition and become more dynamically unstable, reduced tubulin palmitoylation may be correlated with the dynamic-unstable property of MT during mitosis. We thus hypothesized that TOFA-induced perturbations in tubulin palmitoylation may alter MT dynamic instability to induce spindle defects.

### Altering tubulin palmitoylation disrupts MT dynamic instability

**Fig. 6:**
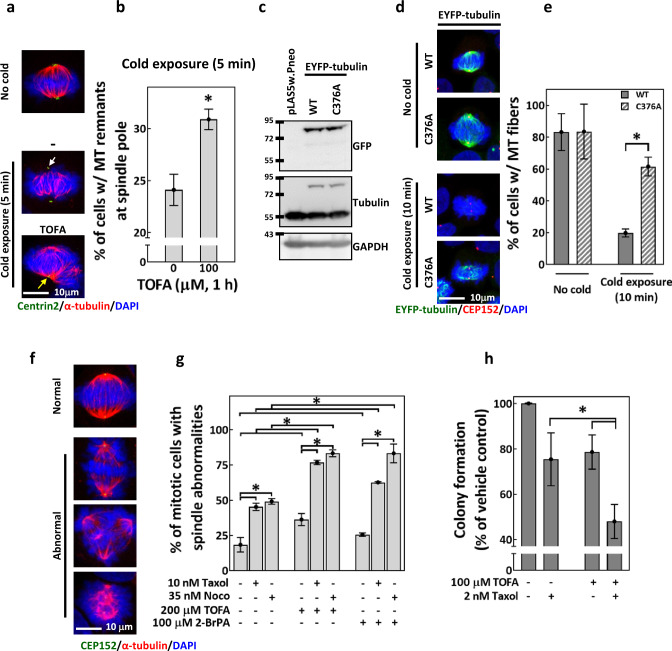
TOFA and palmitoylation-deficient tubulin alter MT dynamic instability. **a**, **b** TOFA hampered cold-induced MT disassembly at the SP. **a** Representative images of cells subjected to cold exposure. Cells were untreated (−) or pretreated for 1 h with 100 μM TOFA, followed by 5 min cold exposure before fixation and staining for centrin2 (green), α-tubulin (red), and DAPI (blue). Cells fixed without cold exposure (No cold) served as the control. The white arrow indicates the site of MT disassembly at the SP, and the yellow arrow indicates the site where MT remnants were observed at the SP after cold exposure. **b** Percentages of untreated or TOFA-pretreated CGL2 cells with MT remnants at the SP after 5 min cold exposure. Mean ± SD from three independent experiments is shown. *P < 0.05 by Student’s *t* test. **c**–**e** C376A mutation on α-tubulin renders spindle MTs resistant to cold-induced disassembly. **c** Western blots show the expression efficiency of EYFP-tubulin-WT and -C376A in HeLaS3 cells. Cells transduced with empty vector pLAS5w.Pneo served as the control. **d** Representative images of EYFP-tubulin-WT- or C376A-expressing cells subjected to 10 min cold exposure, fixation, and staining for CEP152 (red) and DAPI (blue). MT fibers of EYFP-tubulin are displayed in green. Cells fixed without cold exposure (No cold) served as the control. **e** Percentages of the cells described in (**d**) that exhibit MT fibers with EYFP-tubulin-WT and -C376A. Mean ± SD from two independent experiments is shown. *P < 0.05 by Student’s *t* test. **f** Representative image of the normal and abnormal mitotic spindles. Cells were stained for CEP152 (green), α-tubulin (red) and DAPI (blue). **g** TOFA and 2-BrPA enhanced taxol- and nocodazole-induced spindle abnormalities. CGL2 cells were treated for 4 h with TOFA or 2-BrPA, alone or in combination with 0.5-h treatments of taxol or nocodazole as indicated. Treated cells were fixed and stained for spindle analysis. Percentages of cells with abnormal spindles (as in **f**) are shown as mean ± SD of at least 600 cells from two independent experiments. *P < 0.05 by Student’s *t* test. **h** TOFA enhanced taxol-induced cytotoxicity. MDA-MB-231 cells were treated with TOFA and taxol for 24 h, alone or in combination as indicated. Treated cells were subjected to colony-formation assay. Percentages of colonies formed compared to the vehicle control are shown as mean ± SD from six independent experiments. *P < 0.05 by Student’s *t* test.

To validate our hypothesis, we subjected untreated or TOFA-pretreated CGL2 cells to the cold exposure assay, which disassembles MTs at the SP. As shown in Fig. 6a, b, 5-min cold exposure disassembled MTs less efficiently after 1-h TOFA pretreatment. These suggested that TOFA, treated at a concentration that decreases tubulin palmitoylation, reduced cold-induced MT disassembly at the SP. We also examined the effects of cold exposure to MT fibers comprised of the EYFP-fused tubulin-C376A (in which Cys^376^, the proposed site of palmitoylation [45], is substituted with an palmitoylation-deficient alanine residue [46]). EFYP-tubulin-wild-type (WT) and -C376A were expressed in HeLaS3 cells (Fig. 6c) and formed polymerized MT fibers in 80% of the mitotic cells (Fig. 6d, e, no cold). After 10-min cold exposure, the mitotic cells exhibited MT fibers were approximately 20% in EYFP-tubulin-WT-expressing cells and significantly increased to 60% in EYFP-tubulin-C376A-expressing cells (Fig. 6d, e, cold exposure), suggesting that palmitoylation-deficient tubulin rendered MT fibers resistant to cold-induced disassembly. We concluded that proper regulation of tubulin palmitoylation is required for accurate MT dynamic instability during mitosis; ACC inhibition by TOFA may perturb tubulin palmitoylation and thus disrupt the control of MT dynamic instability.

Since TOFA disrupted tubulin palmitoylation and MT dynamic instability, we tested if it can affect the mitotic and cytotoxic effects of MT-targeting drugs. We found that the spindle defects induced by taxol or nocodazole alone can be further increased by pretreatment of TOFA (Fig. 6f, g, TOFA). 2-BrPA pretreatment also further increased taxol- and nocodazole-induced spindle defects (Fig. 6g, 2-BrPA). These data suggest that disruption in FA synthesis and protein palmitoylation may enhance the effects of taxol and nocodazole on spindle assembly. TOFA also enhances the cytotoxicity of taxol in MDA-MB-231 cells (Fig. 6h). Together, our data indicated that TOFA disrupts the dynamic regulation of tubulin palmitoylation, perturbs the control of MT dynamic instability and enhances MT-targeting drugs-induced spindle abnormalities and cytotoxicity.

## Discussion

The tubulin PTMs play essential roles in cell shaping, movement, division and intracellular transport. Thus, it may participate in various MT-related human pathologies [1–3, 5]. In addition, regulation of FA metabolism can regulate the development of MT-based structures [47, 48]. In this study, we found that appropriate regulation of ACC is critical during mitosis to ensure faithful mitotic spindle assembly, potentially by controlling the level of tubulin palmitoylation.

Previously, fission yeasts mutated at *cut6* and *lsd1/fas2* (the respective homologs of ACC and FASN) were shown to exhibit the defective “cut” phenotype during cytokinesis, in which the nucleus is intersected by the medial septum, potentially due to reduced phospholipid production and failed nuclear envelope expansion [20, 22]. These findings implied that delicate regulation of FA synthesis is required for proper membrane dynamics/organization during cytokinesis to ensure faithful mitosis progression. In mammalian cells, pSer^79^-ACC was found localized at the SP from prophase through early anaphase [24, 25] and that acute inhibition of PLK1, a master mitotic kinase, abrogates SP-localization of pSer^79^-ACC [25]. These findings suggest a mitosis-specific phosphorylation of ACC and imply that FA synthesis in mammalian cells may also be regulated in early mitosis, when spindle assembly is initiated. However, the mechanism through which ACC contributes to mitotic progression in mammalian cells has not been previously elucidated. Our observations that increased pSer^79^-ACC level at the SP of mitotic cells is acutely altered by Ro-3306, AICAR and compound C confirmed previous findings regarding mitosis-specific ACC phosphorylation. Furthermore, altering pSer^79^-ACC level by ACICAR and compound C, disrupting ACC activity by TOFA, and depleting ACC protein by shRNAs all increased mitotic spindle abnormalities. These data suggested that precise ACC activity and thus tightly regulated FA synthesis are required for mitotic spindle assembly and may partially explain how FA synthesis affects mitosis progression Notably, *Drosophila* germline lacking ACC also exhibited mitotic spindle abnormalities, indicating that the tight regulation of FA synthesis (and ACC) during spindle assembly may be conserved across different organisms and may be critical for physiological processes that involve mitotic cell division, such as germ cell division and differentiation.

The downstream effects of disrupted FA synthesis include decreased palmitate production [49, 50], upregulated FA β-oxidation [26, 27], and increased protein acetylation [28, 29, 35]. Since the TOFA-induced spindle defects were rescued by exogenous PA, but not β-oxidation inhibitor (etomoxir) or protein acetylation-disrupting agents (C646 and SB204990), our data revealed a plausible role for palmitate to mediate the effects of ACC on spindle assembly. Furthermore, 2-BrPA, ML348, ML349, and palmostatin B also acutely induced spindle defects, indicating that protein palmitoylation, one of the critical functions of palmitate [38], may be tightly regulated during mitosis to ensure spindle assembly. Accordingly, the human palmitoyl-proteome (available from the SwissPalm database) includes several mitotic proteins, such as centrosome components and MT motors [51]. Collectively, we reason that precise control of ACC activity and tightly regulated FA synthesis ensure accurate spindle assembly by controlling protein palmitoylation during mitosis.

The various PTMs of tubulin play critical roles in the properties, functions, and dynamics of MTs [1, 2]. Accurate spindle assembly relies on delicate control of MT dynamics and thus also requires precise spatiotemporal regulation of tubulin PTMs [4]. Moreover, previous studies have shown that tubulin is subjected to palmitoylation in yeast [10], rodent cells [7, 8], human platelets [9], and in proliferating cancer cells [50]. Except for mediating MT-membrane association [8, 9], the cellular and physiological impacts of tubulin palmitoylation remain elusive. We found in this study that tubulin palmitoylation is reduced during mitosis, coincident with the dramatic changes in cell shape and MT dynamic instability after mitosis entry. Similarly, tubulin palmitoylation was shown reduced after platelet activation [9], a process that also involves dramatic changes in cell shape and MT dynamics/organization [52]. These findings imply the correlations of tubulin palmitoylation level with MT dynamic instability. We therefore speculate that during mitosis, tubulin may undergo a dynamic palmitoylation/depalmitoylation cycle to allow for intricate control of MT dynamic instability, which is an essential MT property for accurate spindle assembly. Notably, our data showed that a TOFA treatment regimen that reduces tubulin palmitoylation is also sufficient to suppress cold-induced MT disassembly, induce spindle abnormalities and enhance nocodazole- or taxol-induced effects. Consistently, MT fibers comprised of palmitoylation-deficient mutant tubulins are more resistant to cold-induced disassembly than those comprised of WT tubulin. These findings support the idea that regulated tubulin palmitoylation is an essential factor of MT dynamic instability. Furthermore, TOFA may act to limit tubulin (re)palmitoylation during mitosis, thereby disrupting the tubulin palmitoylation/depalmitoylation cycle and causing defects in MT dynamic instability and spindle assembly.

A previous report showed that pharmacological AMPK activation and the subsequent ACC inhibition can rescue defective MT-dependent cholesterol transport and MT reformation after recovery from cold treatment in cystic fibrosis cell lines [53], suggesting that AMPK-ACC axis may regulate MT functions and dynamics. Another study demonstrated that AMPK is activated during FA starvation to reorganize detyrosinated MTs through an unknown mechanism; this reorganization promotes lipid droplet (LD) dispersion on the detyrosinated MTs, thereby increasing LD-mitochondria contacts to upregulate FA β-oxidation [54]. These findings implied that pathophysiological alterations in lipogenesis pathways may control MT functions by modulating tubulin PTMs, thus reprogramming cellular functions to meet metabolic demands. In this study, we found that the phosphorylation of AMPK and ACC at mitotic entry occurs concomitantly with a physiological reduction of tubulin palmitoylation. In addition, ACC inhibition by TOFA disrupt MT dynamic instability and induce spindle defects. Based on these findings, we hypothesized that the Ser^79^-phosphorylation of ACC at mitotic entry may contribute to the stringent regulation of lipogenesis, reducing palmitate production to limit tubulin palmitoylation during mitotic entry until metaphase. This regulatory mechanism may serve to precisely manage MT dynamic instability and control spindle assembly. Our data thus reveal a model that coordination between the lipogenesis pathway and MT functions supports spindle assembly and mitosis progression, in particular by modulating tubulin modifications.

Our results showed that TOFA may induce spindle defects by disrupting tubulin palmitoylation and MT dynamic instability and can enhance taxol-induced spindle abnormalities and cytotoxicity. These are consistent with a previous study showing that FASN inhibitors enhance the antiproliferative efficacy of taxane [50] and imply that disruption of FA synthesis may enhance the anticancer effect of taxol. Since upregulated ACC and FASN and deregulated protein palmitoylation are associated with many types of cancer, modulating FA synthesis pathway and protein palmitoylation have been considered as plausible anticancer strategies [14, 15, 17, 18]. Our findings thus expand the understanding of how interfering FA synthesis could benefit the anticancer effects of anti-MT drugs. Future studies regarding how overexpression of ACC and FASN affects the tubulin palmitoylation and MT functions would provide further insights into such anticancer strategies.

## Materials and methods

### Cell culture and drug treatments

CGL2 cells (the hybrid of HeLa cell and human fibroblast), MDA-MB-231 cells and HeLaS3 cells were obtained and cultured as previously described [55, 56]. The drugs and chemicals used in this study include: CDK1 inhibitor, Ro-3306 (No. 15149, Cayman Chemical, Ann Arbor, MI, USA); AMPK activator, AICAR (No. 10010241, Cayman); AMPK inhibitor, compound C (No. 171260, Merck, Darmstadt, Germany); 5-(tetradecyloxy)-2furancaboxylic acid (TOFA, No. 10005263, Cayman); palmitic acid (PA, P5585, Sigma Aldrich, St. Louis, MO, USA); FA β-oxidation inhibitor, (+)-etomoxir (No. 11969, Cayman); protein acetylation inhibitor, C646 (No. 10549, Cayman); ATP-citrate lyase inhibitor, SB204990 (No. 154566-12-8, Cayman); protein palmitoylation inhibitor, 2-bromopalmitic acid (2-BrPA; #21604, Sigma); protein depalmitoylation inhibitors, ML348 (No. 18523, Cayman), ML349 (No. 20923, Cayman) and palmostatin B (#508738, Merck); tubulin-targeting drugs, taxol (paclitaxel, 580555, Merck), and nocodazole (V1377, Sigma).

Cells were subjected to double-thymidine block and release as previously described [57] to enrich populations at each cell cycle stage; attached cells at 0, 3, 6, and 9 h after thymidine release were collected as G1, early S, late S, and G2 cells, respectively, and the floating cells at 9 h were collected as the mitotic cells. Alternatively, the cell cycle was blocked by 14–16 h treatment with 2–5 μM Ro-3306 (optimal duration and concentration were empirically determined for each cell line), followed by a PBS wash and incubation in drug-free medium for 30–45 min to release the block and allow mitotic entry for collecting mitotic cells [58]. The mitotic cells then were fixed for staining or shaken off the plates and collected. The cytotoxicity of each drug was tested for each cell line with the trypan blue exclusion assay or colony-formation assay as previously described [56], in order to empirically determine the appropriate treatment concentration.

### Depletion of cellular ACC

The shRNAs targeting ACC1 (gene symbol *ACACA*, TRCN-232456, and -232458) and ACC2 (gene symbol *ACACB*, TRCN-3093, and -10759) were purchased from the National RNAi Core Facility (Genomic Research Center, Academia Sinica) and were used to deplete ACC proteins in cell culture. The shRNA-containing virions were prepared, and transient depletion of endogenous ACC were performed as described previously [55]. Empty vector pLKO.1-containing virions were also prepared as the control.

### Examination of mitotic spindles in ACC RNAi *Drosophila* germarium

The following fly lines were obtained from Bloomington stock center. The *nanos (nos)-Vp16-GAL4* line (#4937) and *ACC RNAi* line (#34885) were crossed to generate flies (*nos* > *ACC*^*RNAi*^) with ACC knockdown in the germline. The crosses were cultured at 18 °C to reduce GAL4 activity to avoid developmental influence. *nos* > *mCherry*^*RNAi*^ flies generated from the cross of *nos-Vp16-GAL4* line and *mCherry RNAi* line (#35785) were used as the control. The experiment was performed with two biological replicates. Ten newly eclosed females with the indicated genotypes were randomly picked from a standard cross and shifted to 29 °C for 7 days; ovaries were dissected and subjected to immunostaining. Ovaries from at least ten pairs of ovaries were separated, mixed, and mounted for observation; 400 germaria were examined. Immunostaining of the *Drosophila* germaria was performed as previously described [59]. The antibodies included: anti-ACC, a gift from Dr. Jacques Montagne (1:100; French National Centre for Scientific Research, France) described previously [60], anti-1B1 (1:25; Developmental Studies Hybridoma Bank, DSHB), anti-α-tubulin (1:100; GTX112141, GeneTex Hsinchu, Taiwan, or T5168, Sigma) and anti-γ-tubulin (1:250; T3559 or T6557, Sigma).

### Immunofluorescence staining and confocal microscopy of cultured cells

Cells seeded on coverslips were fixed and subjected to immunofluorescence staining as previously described [55]. The primary antibodies included anti-ACC (#3662, Cell Signaling, Danvers, MA, USA), anti-pSer^79^-ACC (#3661, Cell Signaling), anti-FASN (#3180, Cell Signaling), anti-AMPKα2 (GTX13487, GeneTex), anti-pThr^172^-AMPK (#2535, Cell Signaling), anti-α-tubulin (GeneTex or Sigma), anti-CEP152 (ab183911, Abcam, Cambridge, UK), and anti-Centrin2 (#04-1624, Merck). AlexaFluor 488- and 633-conjugated goat anti-mouse and anti-rabbit IgG were purchased from Invitrogen (Carlsbad, CA, USA). Confocal imaging of the immunostained samples was performed under a Leica TCS-SP5 upright microscope with an HCX PLAPO 63×/1.4 objective at 300 Hz scanning speed; 15–20 μm image stacks were collected with a 0.5-μm step size and were maxima-projected in ImageJ.

For analysis of mitotic spindle abnormalities, the mitotic spindle was revealed by immunostaining for α-tubulin and centrin2 or CEP152, as described above. The numbers of cells with mitotic spindle abnormalities were counted under a Zeiss Axioplan 2 Imaging MOT fluorescence microscope. Mitotic spindles at prophase, prometaphase, and metaphase were evaluated as described previously [55, 57]. Multipolar spindles, disorganized spindles, and bipolar spindles with misaligned chromosomes or with abnormal MT array were regarded as mitotic spindle abnormalities. The level of spindle abnormalities is expressed as the percentage of the mitotic cells containing abnormal spindle.

### Metabolic labeling and visualization of palmitoylated proteins

Metabolic labeling of palmitoylated proteins was performed using palmitic acid-azide (PA-azide, 15-Azidopentadecanoic Acid, C10265, Invitrogen) as described [42]. PA-azide was solubilized in DMSO at 50 mM and kept as a stock solution. The stock was diluted in medium to 100 μM, sonicated at room temperature for 15 min, settled for 15 min, and then used to treat cells seeded on coverslips for 16 h. Cells incubated in PA-azide free medium (No PA) were used as the control. The cells were then fixed in PTEMF buffer [61] for 15 min, washed with PBS, and then subjected to click chemistry using Click-iT Cell Reaction Buffer Kit (C10269, Invitrogen) to conjugate a fluorophore to PA and visualize the palmitoylated proteins. Briefly, the click reaction was performed by immersing cells in the click cocktail (88% reaction buffer, 2% CuSO_4_ and 10% buffer additive) containing 2.5 μM AlexaFluor^TM^ 488-alkyne (A10267, Invitrogen) at a volume of 50 μL/coverslip for 30 min at room temperature. Cells were then washed with PBS and subjected to α-tubulin immunostaining, after which the cells were mounted in Fluoromount-G containing DAPI for confocal imaging.

### Analysis of colocalization between palmitoylated proteins and MTs

Potential tubulin palmitoylation was assessed by examining the colocalization between α-tubulin immunostaining and PA-azide labeled by click chemistry in confocal images. A 24 × 24-μm region of interest (ROI) covering a single mitotic cell and a free-hand drawn ROI covering a single interphase cell were created in ImageJ and were sampled for colocalization analysis in Imaris. The level of colocalization between α-tubulin and palmitoylated proteins in a single cell was assessed by Pearson’s correlation coefficient, the value of which ranges from −1 to 1 and indicates the correlation between two colors above the threshold intensities [62]. The correlations were assessed for 39 interphase cells and 39 mitotic cells collected from two independent experiments.

The colocalization between α-tubulin and click chemistry-labeled palmitoylated proteins was also examined by the proximity ligation assay (PLA), as described previously [42]. After click chemistry-mediated conjugation of AlexaFluor-488-alkyne to the PA-azide, the samples were reacted with anti-α-tubulin (T5168, Sigma) and anti-AlexaFluor-488 (#A-11094, Invitrogen) at 4 °C overnight and then subjected to the PLA procedure using Duolink^®^ In Situ Detection Reagents Red (DUO92008, Sigma) as previously described [43, 57]. Samples were then mounted in Fluoromount-G containing DAPI for observation and imaging. The PLA signals indicate tubulins in a complex with PA at a distance of less than 40 nm. The PLA signal intensity in a single interphase or mitotic cell was measured using Imaris and indicated the level of α-tubulin-PA-azide colocalization.

### Acyl-biotin exchange assay

A previously described [44] procedure for the acyl-biotin exchange assay was adapted in this study to purify the palmitoylated proteins as illustrated in Fig. 5c. Briefly, cycling cells and the Ro-3306-blocked-and-released mitotic cells, with or without TOFA (100 μM, 4 h) treatment, were collected and suspended in lysis buffer (150 mM NaCl, 50 mM Tris-HCl, 5 mM EDTA, 0.2% SDS) supplemented with 5 mg/mL protease and phosphatase inhibitor (Roche), followed by homogenization for 10 min at 4 °C. The homogenates were then incubated for 2 h at room temperature in the presence of 10 mM N-ethylmaleimide (NEM) to block the free thiols. Next, the palmitate moieties linked to cystine residues through thioester linkage in the homogenate were cleaved by the addition of 0.5 M hydroxylamine (HAM) and were replaced with biotin by the addition of EZ-link^TM^ HPDP-biotin (#21341, ThermoFisher, Waltham, MA, USA) (Fig. 5c), followed by overnight incubation of the homogenates at room temperature. Homogenates incubated without HAM were used as the control. The biotinylated proteins were then purified with streptavidin agarose and used for western blotting.

### Analysis of MT dynamic instability and MT nucleation from the SP

A cold exposure assay, in which MT disassembly is induced at the SP, was used to probe the dynamic instability of MTs as previously described [55, 57]. Cells seeded on coverslips were treated with or without TOFA as indicated and then subjected to cold exposure for the indicated time, followed by immunostaining. The percentage of mitotic cells with MT remnants at the SP was calculated for each sample, and differences in the percentages indicated the level of altered MT dynamic instability.

To probe the effects of tubulin palmitoylation on MT dynamic instability, EYFP-tubulin open reading frame in the vector pEYFP-tubulin (#6118-1, BD) was subcloned into pLAS5w.Pneo vector. Cys^376^ in tubulin is the proposed site for palmitoylation [45]. Using site-directed mutagenesis, this residue was substituted with alanine (C376A), which cannot undergo palmitoylation [46]. Virions containing EYFP-tubulin-WT and -C376A were collected and used to transduce HeLaS3 cells as previously described [55]. After transduction, the cells were incubated with 1 mg/mL G418 to establish stable cell lines. HeLaS3 cells stably expressing EYFP-tubulin fusion protein were then seeded on coverslips, subjected to 10 min cold exposure, and analyzed by immunostaining the centrosomes. Percentages of mitotic cells, among those exhibiting fluorescent MT fibers, were calculated. Decreases in the percentages following cold exposure indicated cold-induced disassembly.

### Western blotting

Cell lysis and immunoblotting were performed as described [55]. Proteins were detected using anti-ACC, anti-pSer^79^-ACC, anti-AMPKα2, anti-pThr^172^-AMPK, anti-FASN, anti-Cyclin B1 (sc-245, Santa Cruz, Biotechnology, CA, USA), anti-pHistoneH3 (#9701, Cell Signaling), anti-GFP (G6539, Sigma) and anti-α-tubulin. GAPDH and PCNA were detected with anti-GAPDH (GTX100118, GeneTex) and anti-PCNA (sc-56, Santa Cruz) as the loading controls.

## Supplementary information

Original blots

## Data Availability

The data associated with this study are available from the corresponding author upon request.
