# Supplementary figures and images for: Inhibition of acetyl-CoA carboxylase impaired tubulin palmitoylation and induced spindle abnormalities
Source: Cell Death Discov. 2023 Jan 9;9:4. doi: 10.1038/s41420-023-01301-8 (PMC9826786; doi:10.1038/s41420-023-01301-8)

Fig. 1a

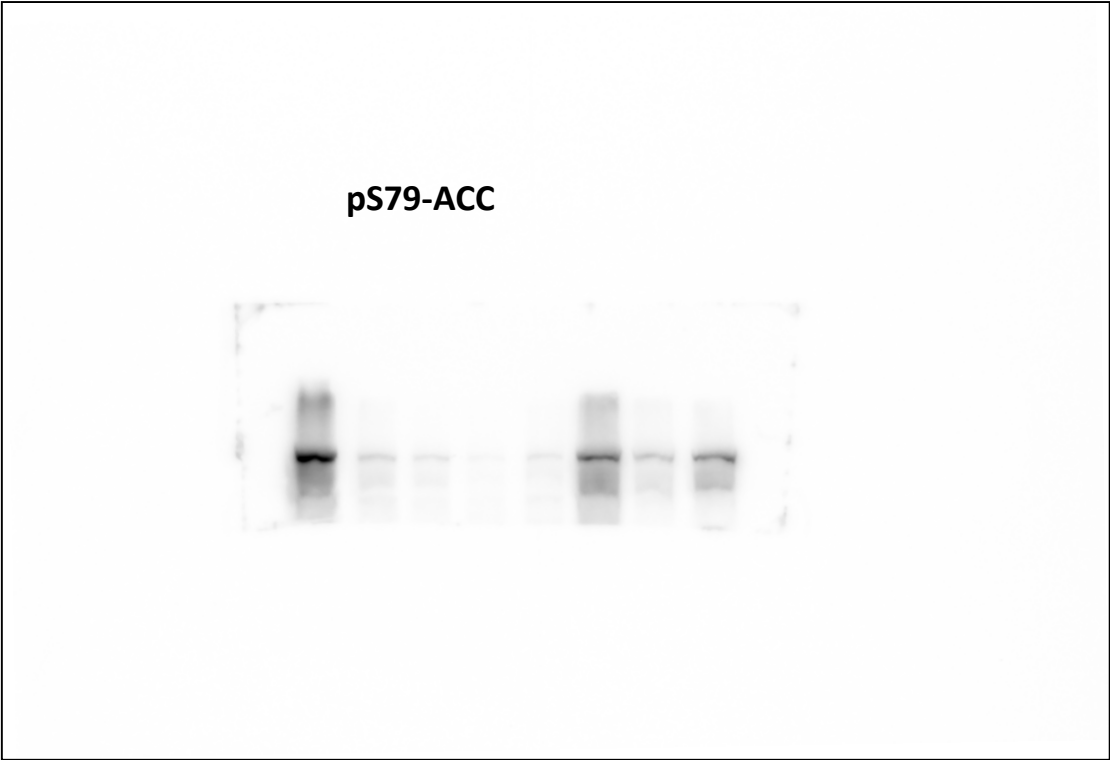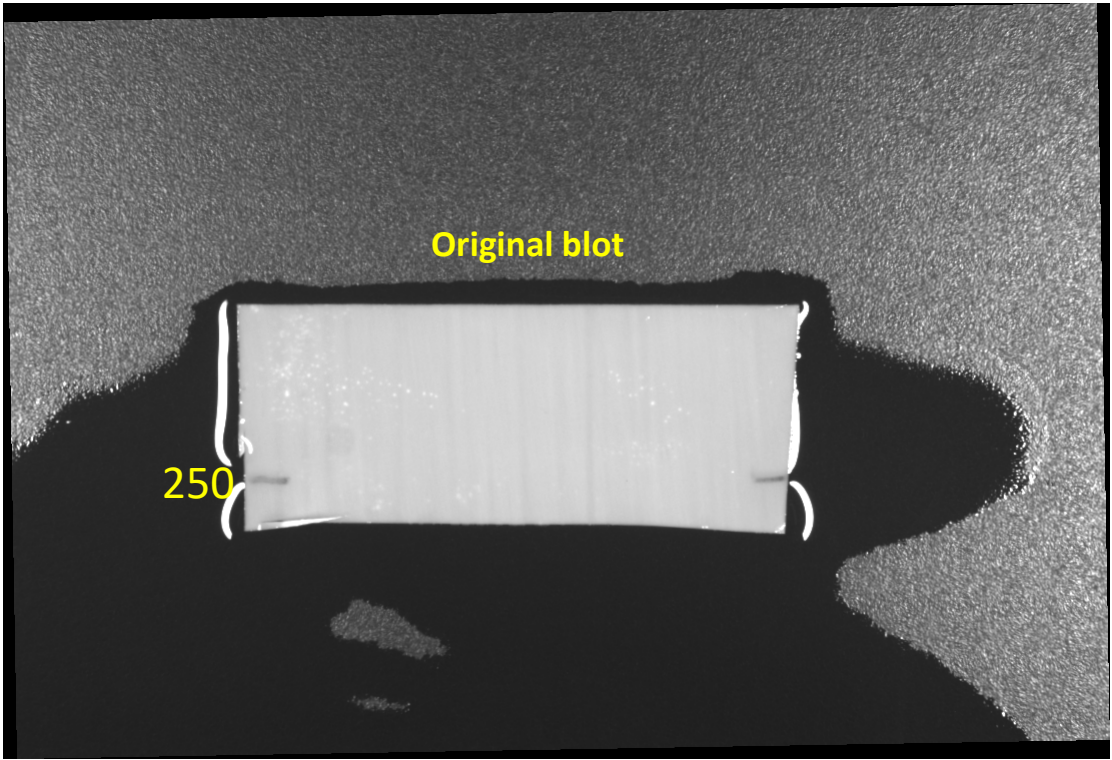

Fig. 1a

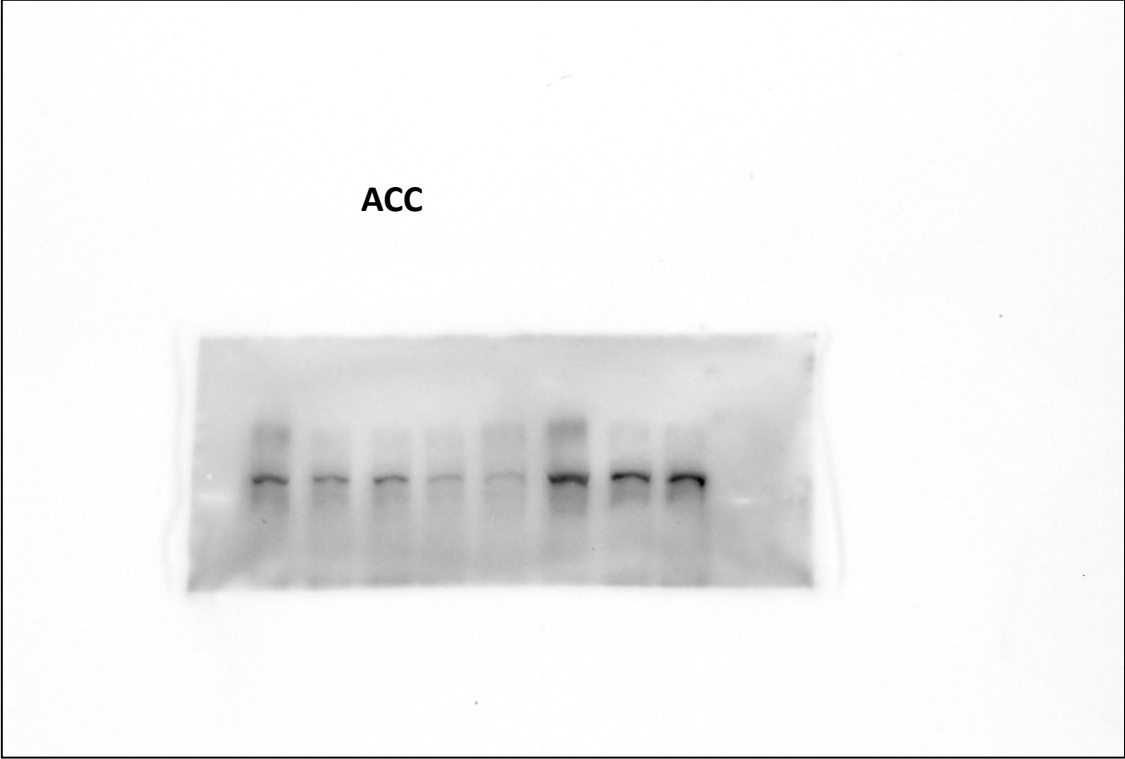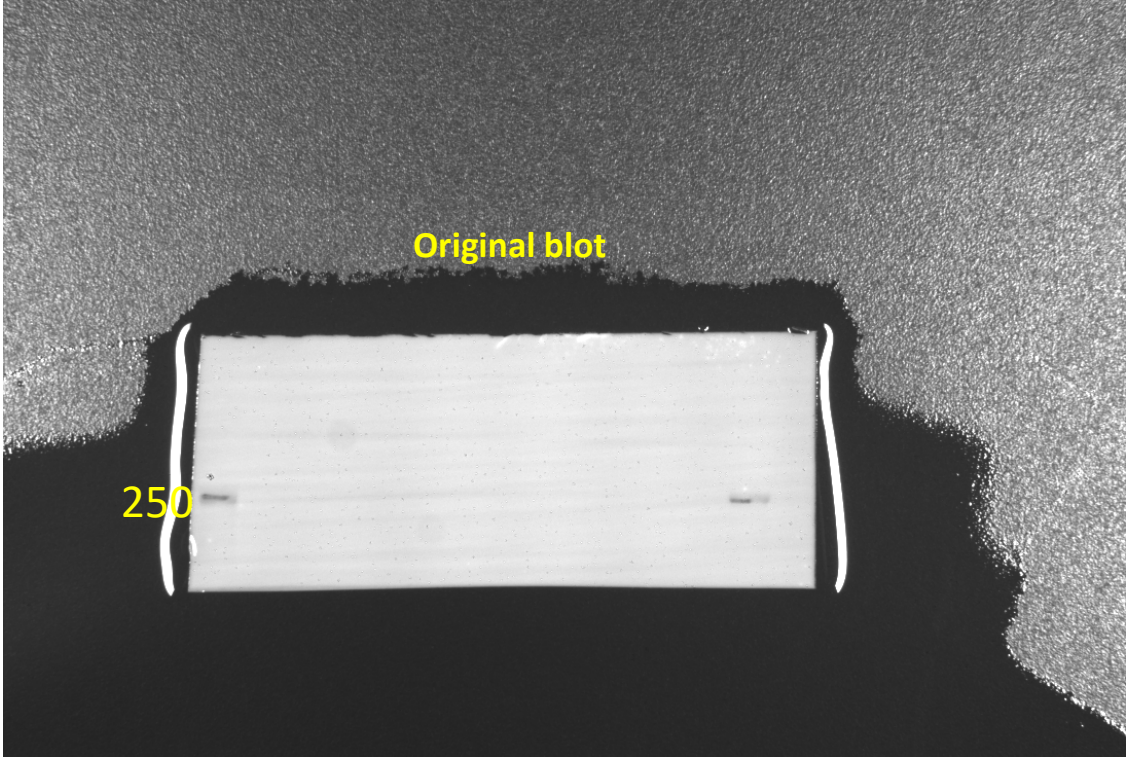

Fig. 1a

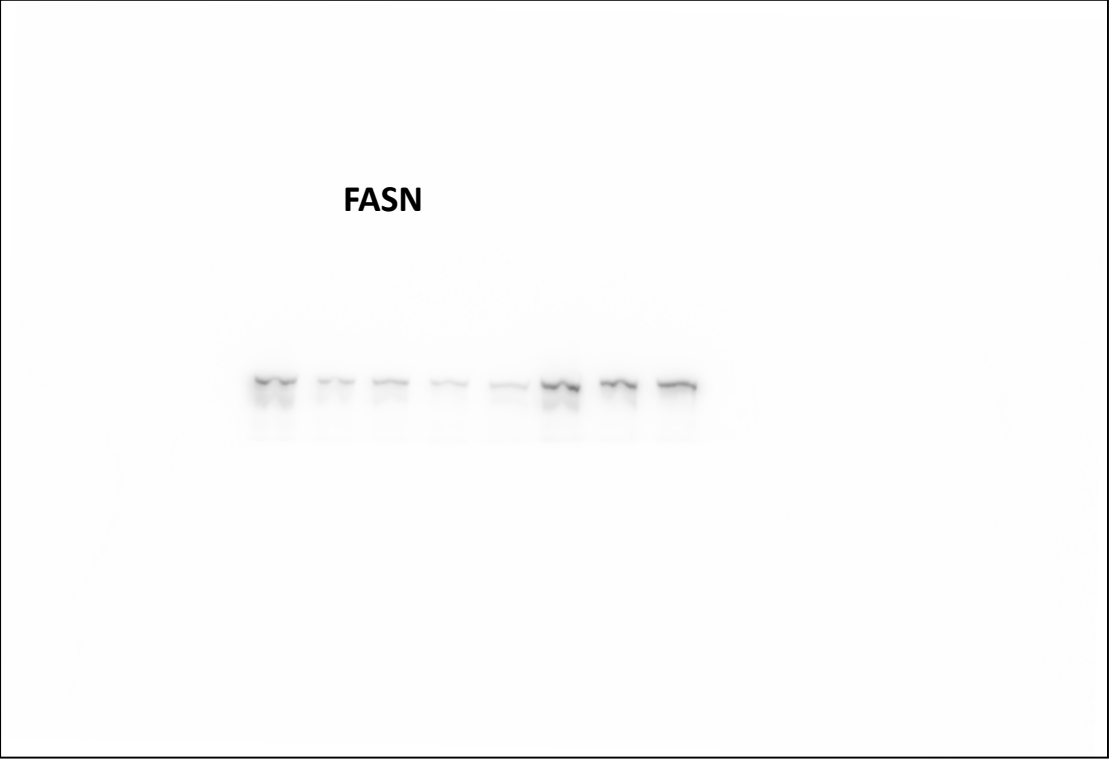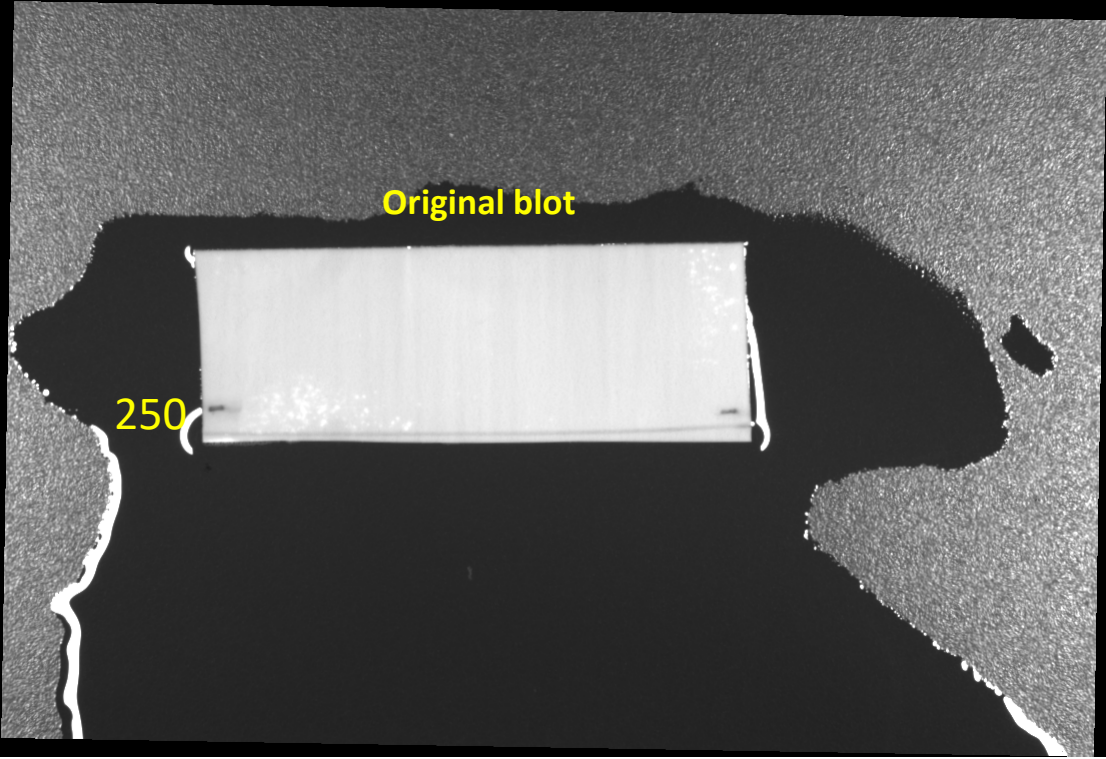

Fig. 1a

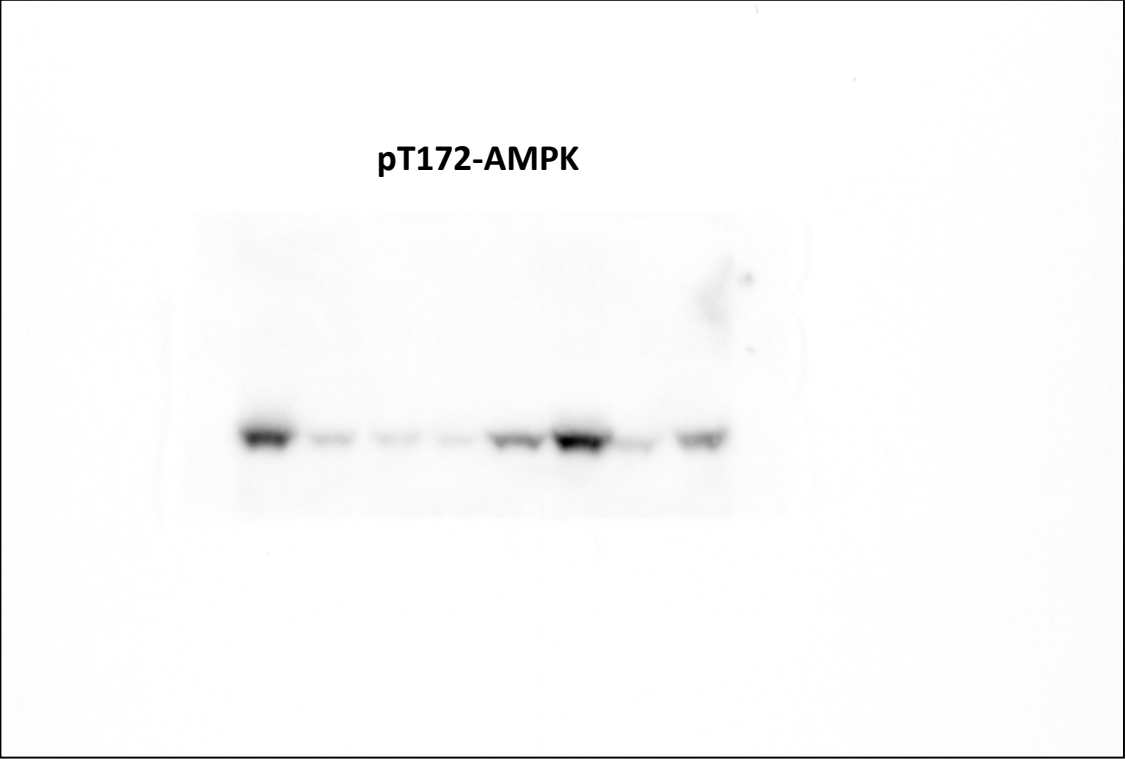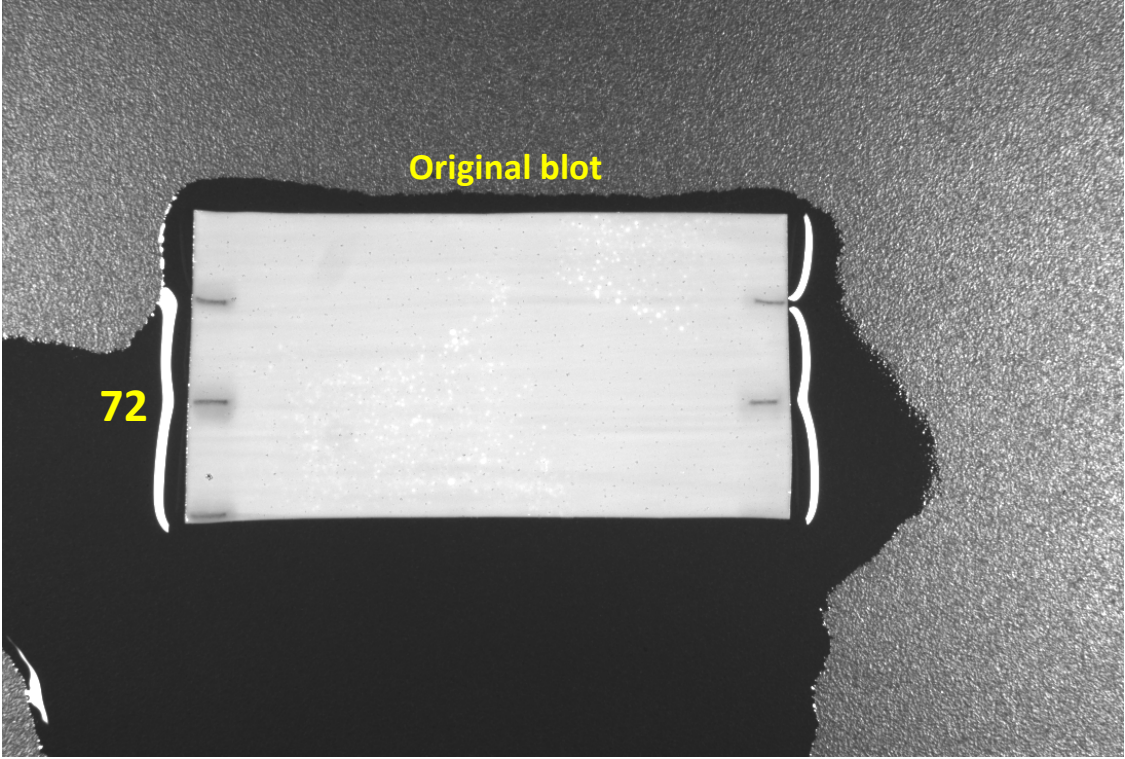

Fig. 1a

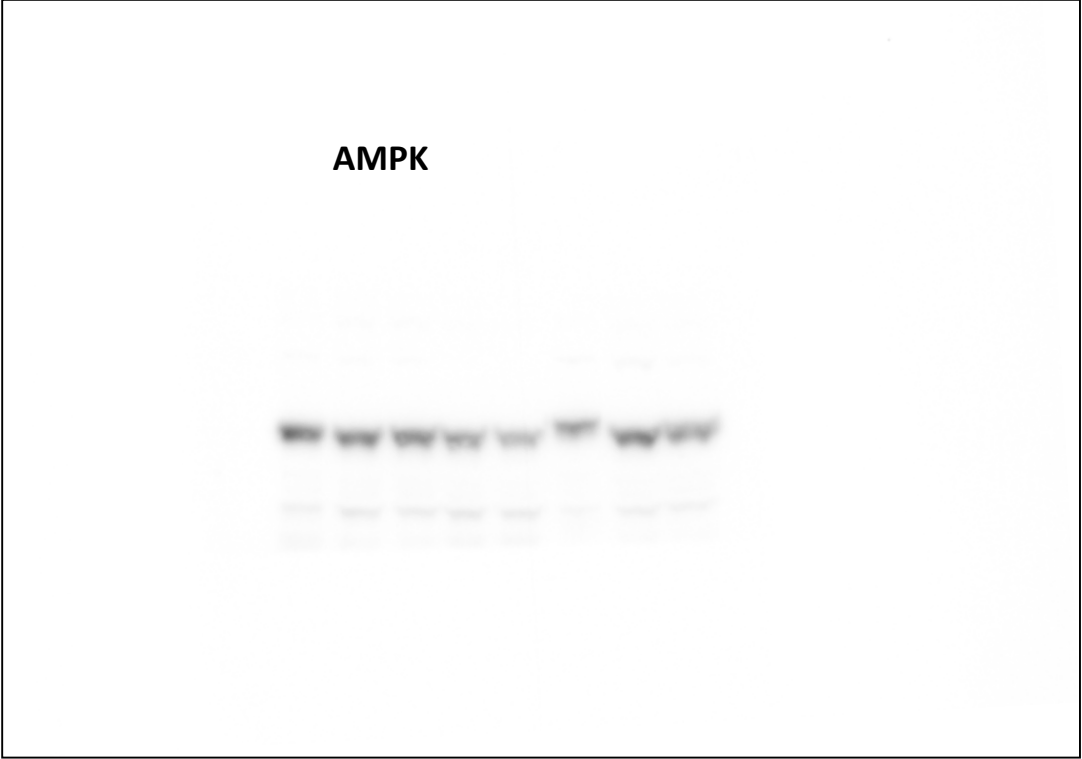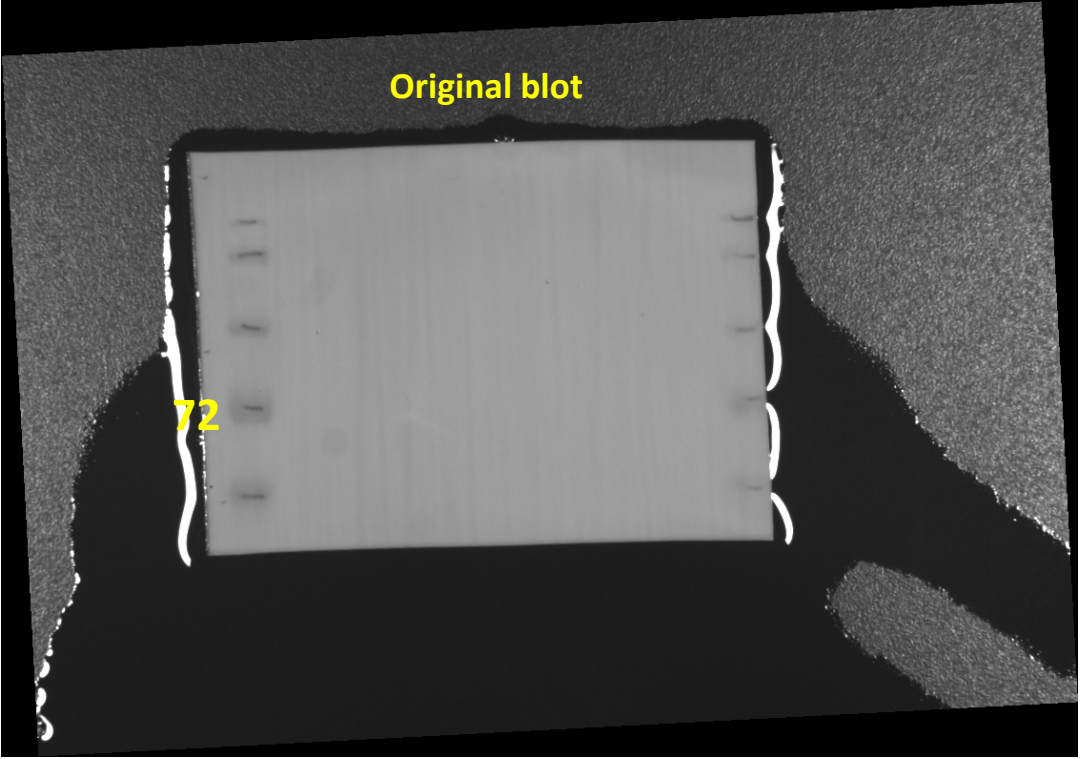

Fig. 1a

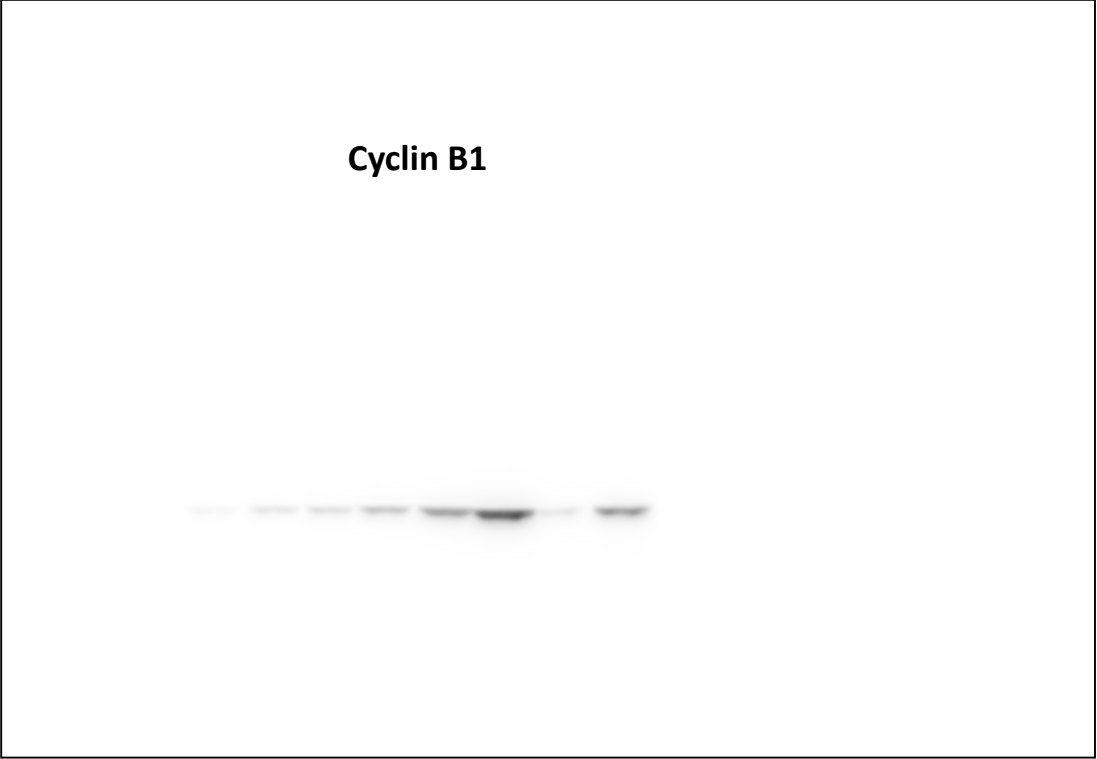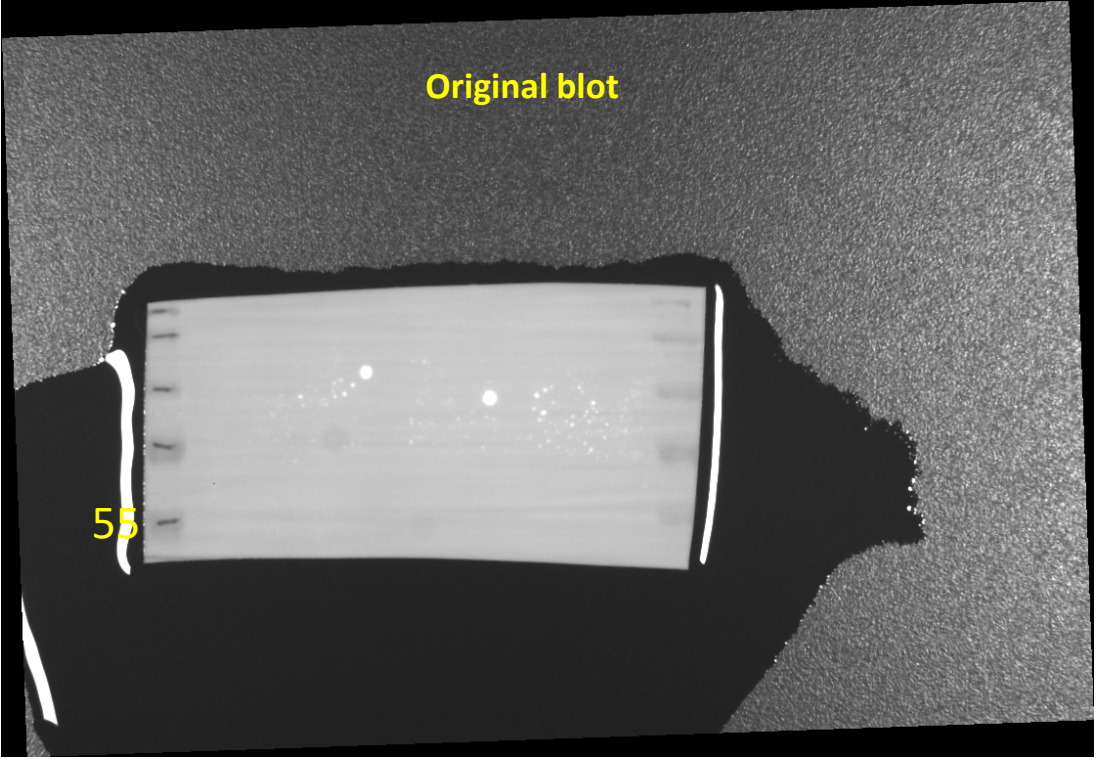

Fig. 1a

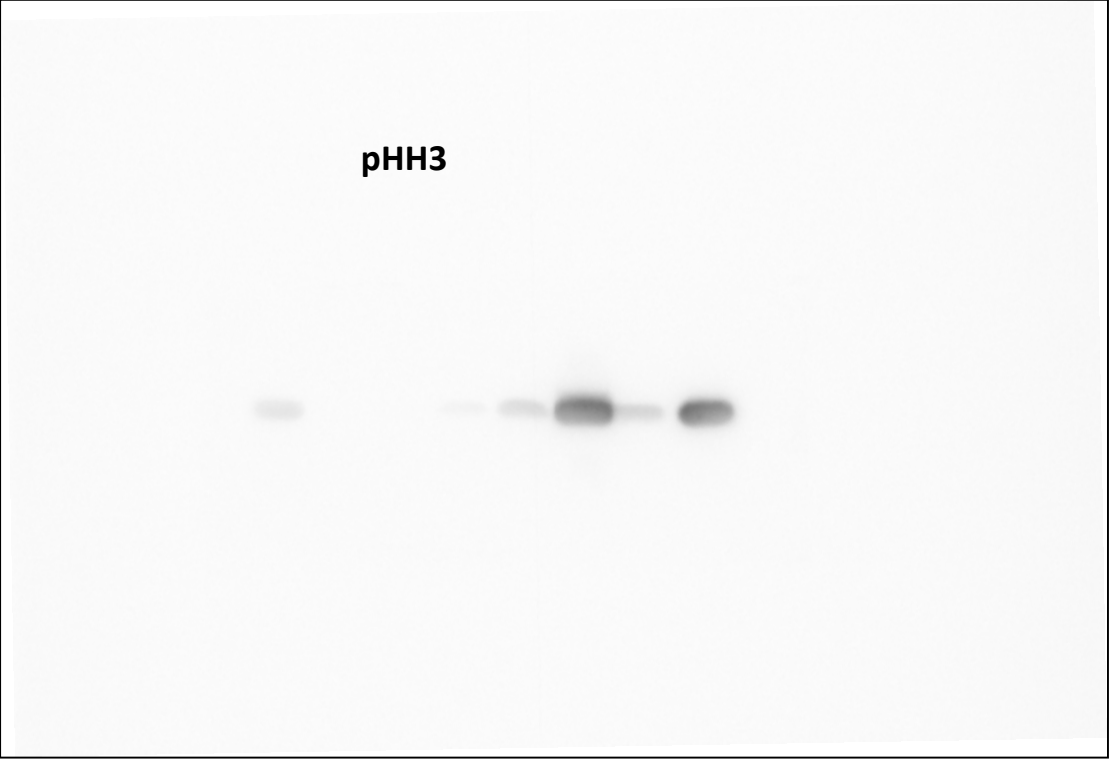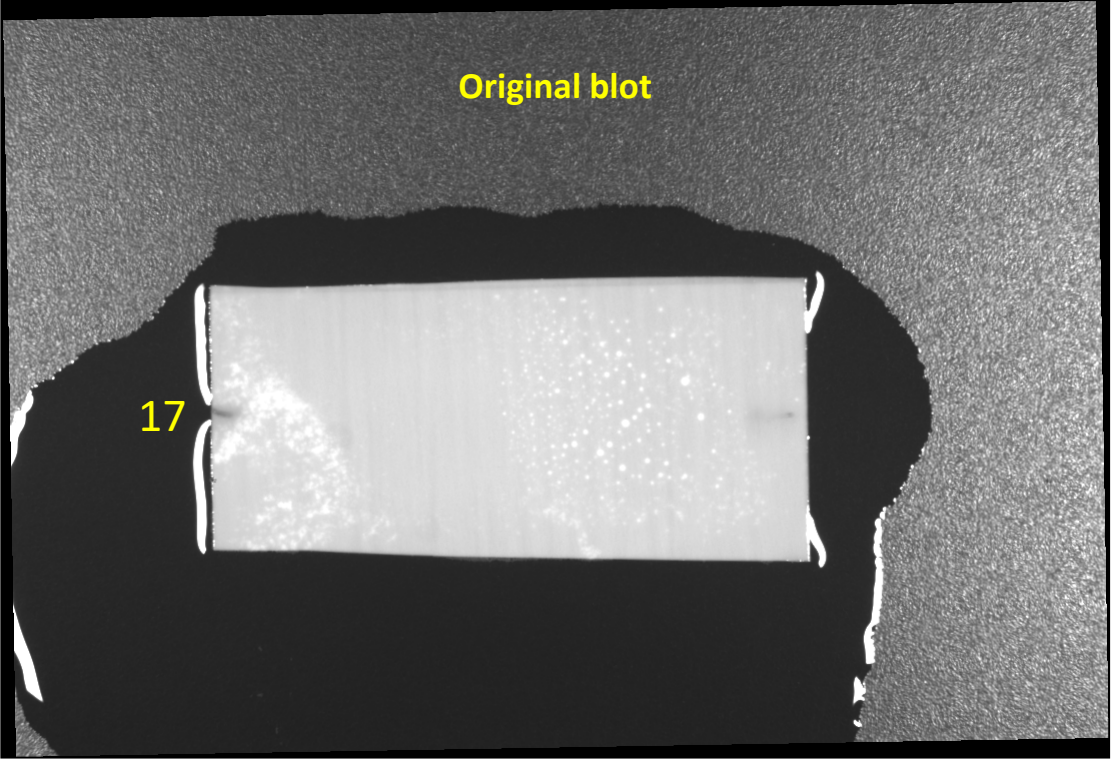

Fig. 1a

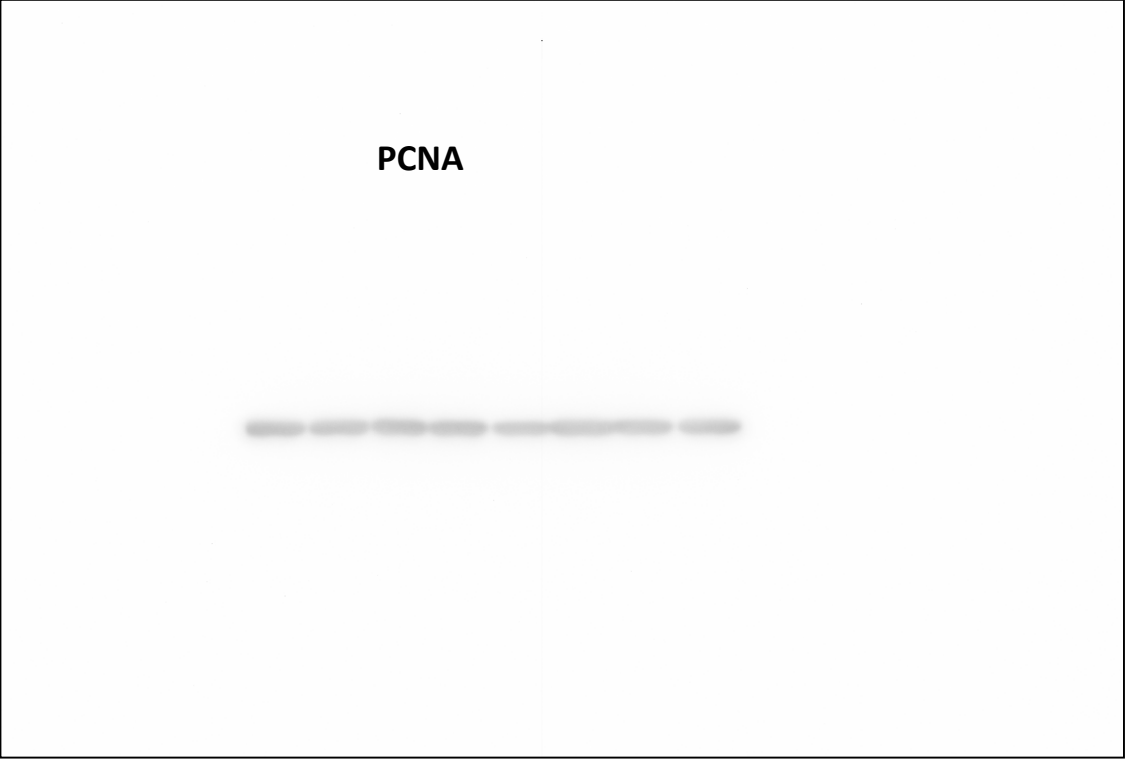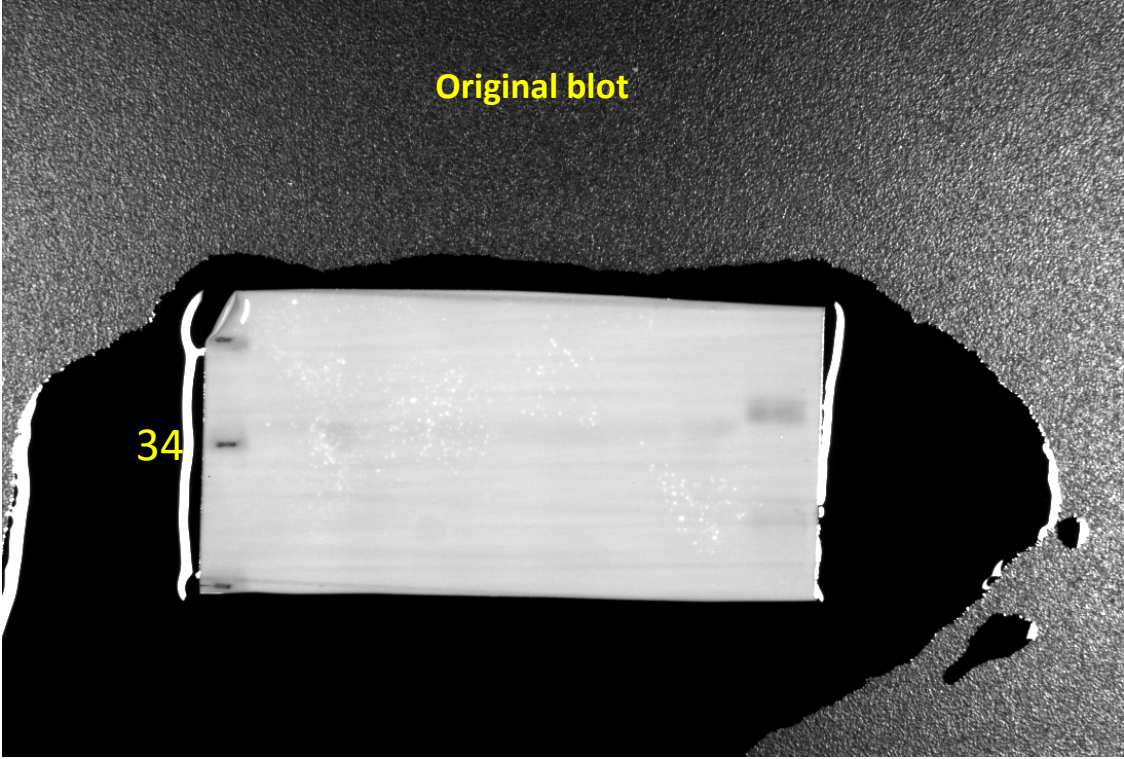

Fig. 1f

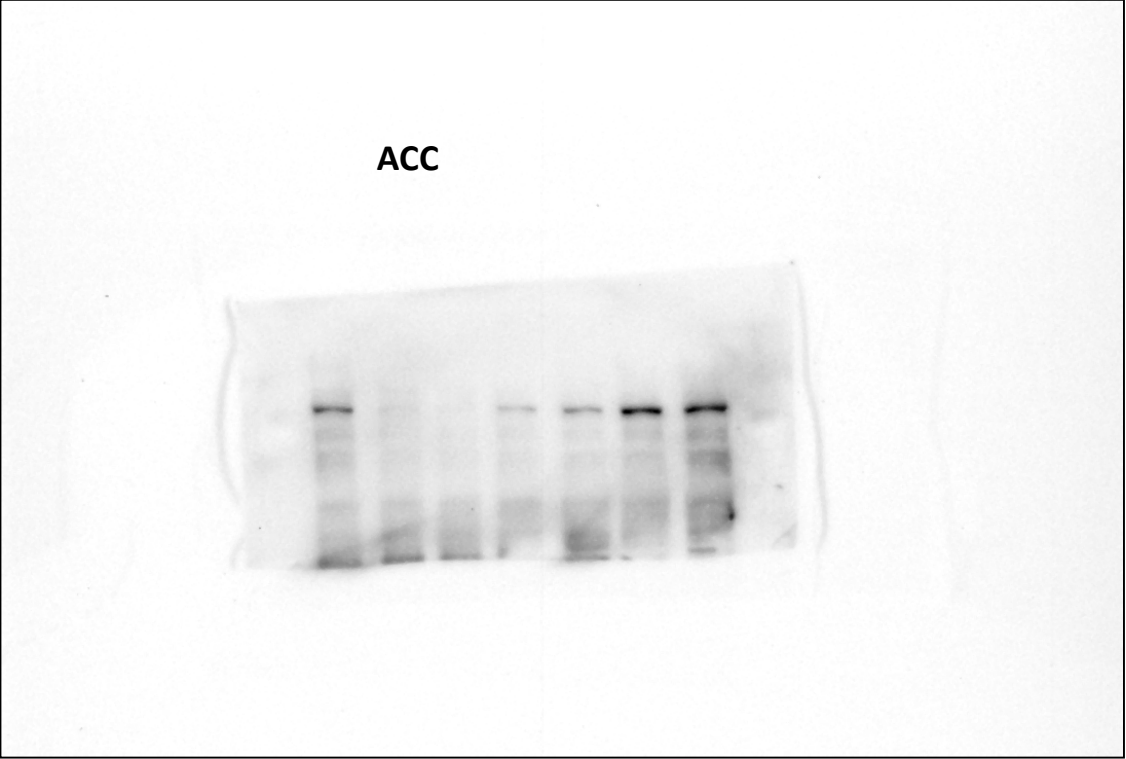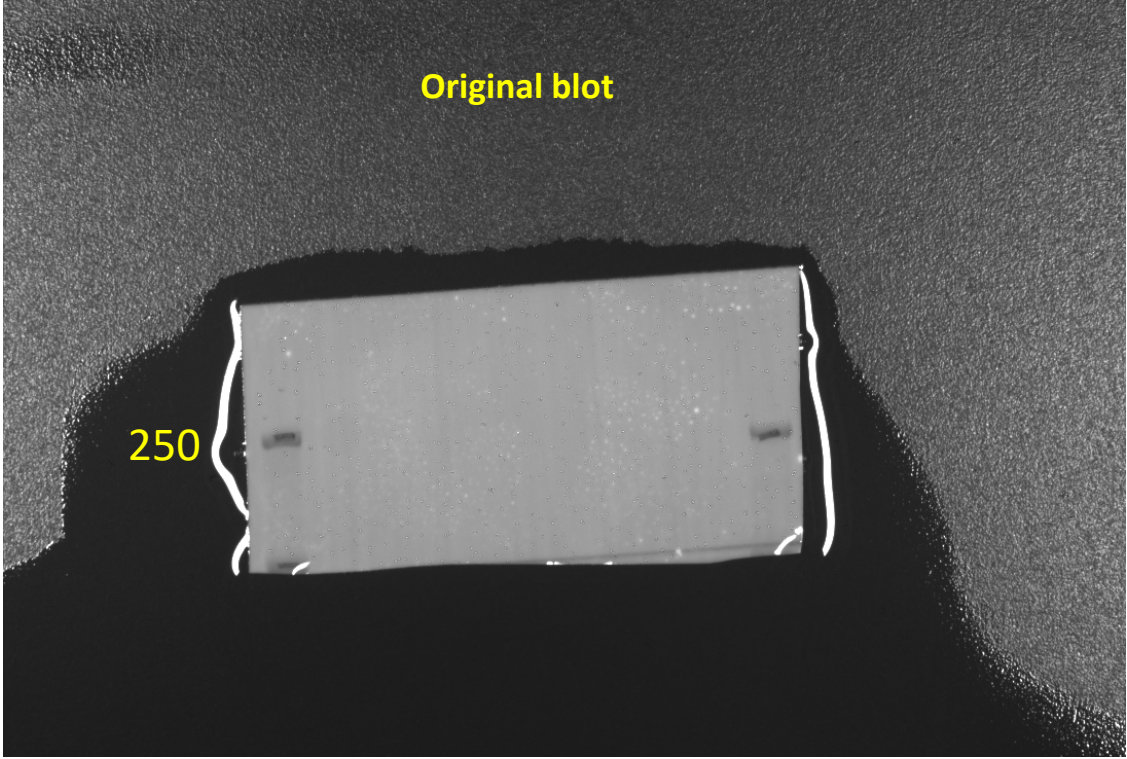

Fig. 1f

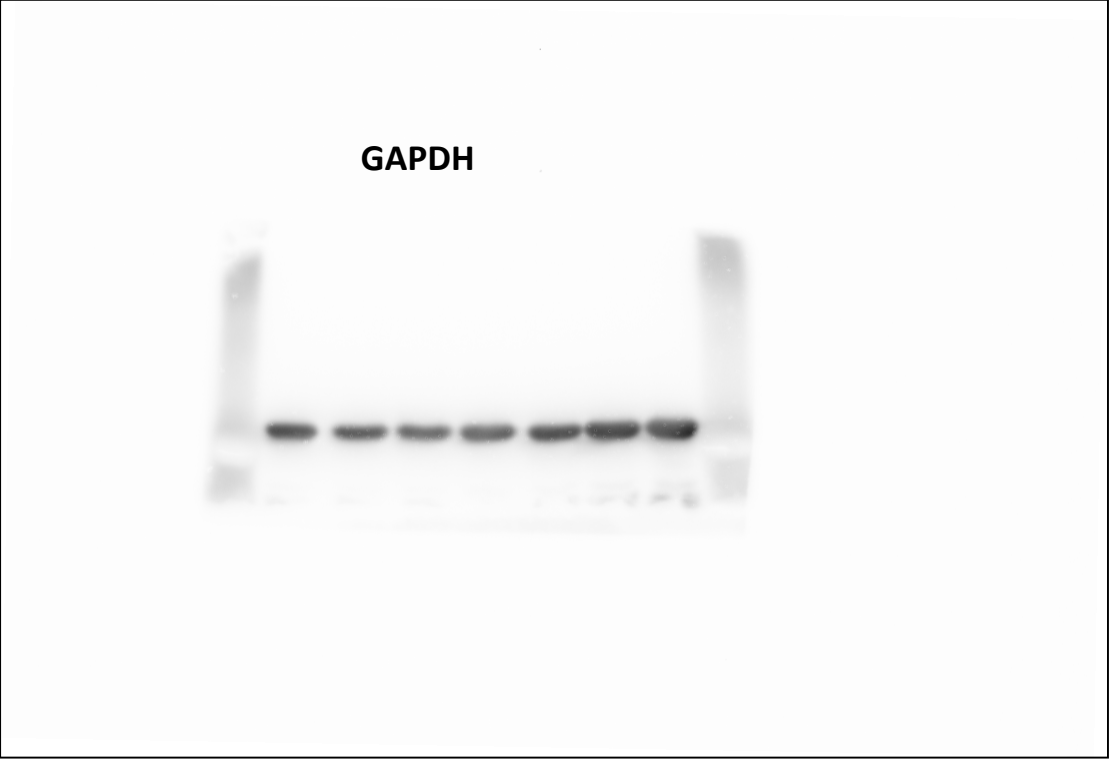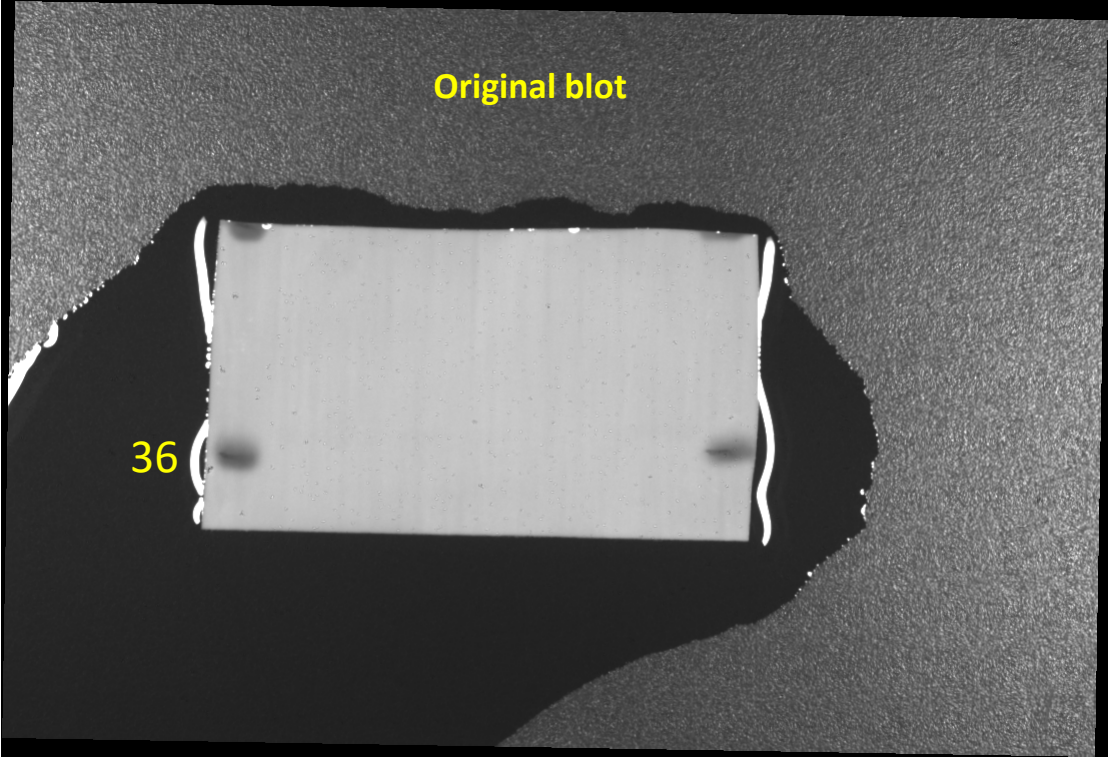

Fig. 5d

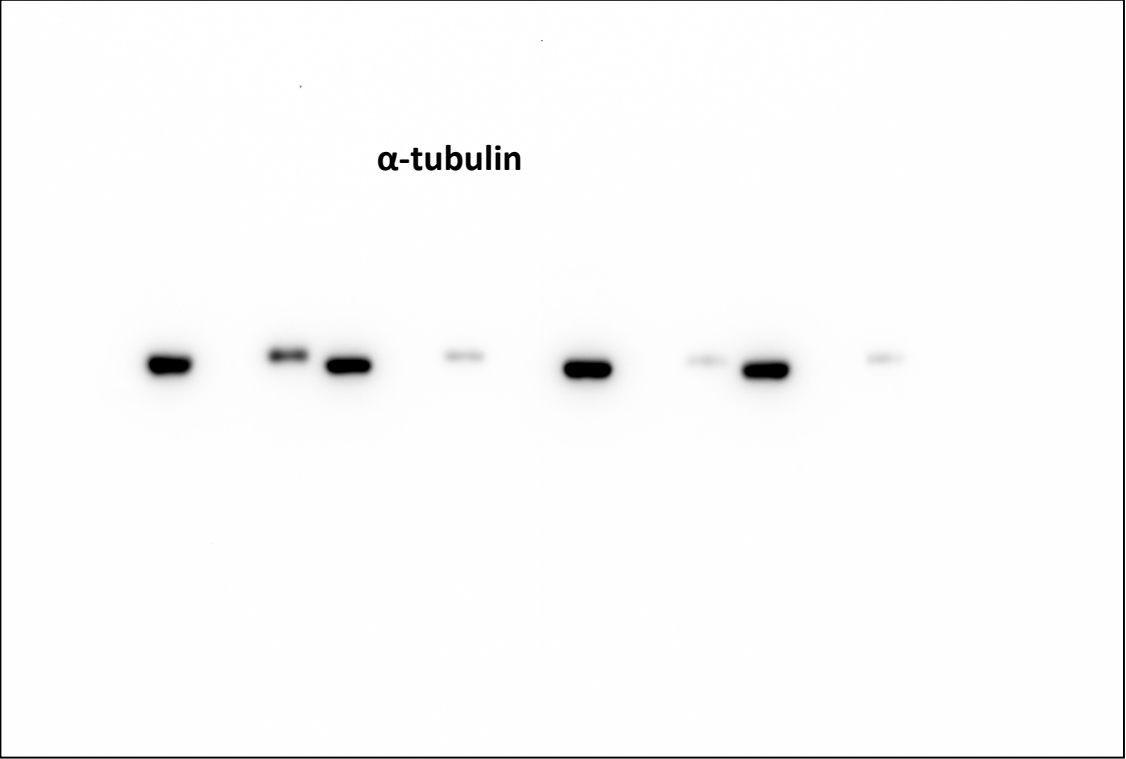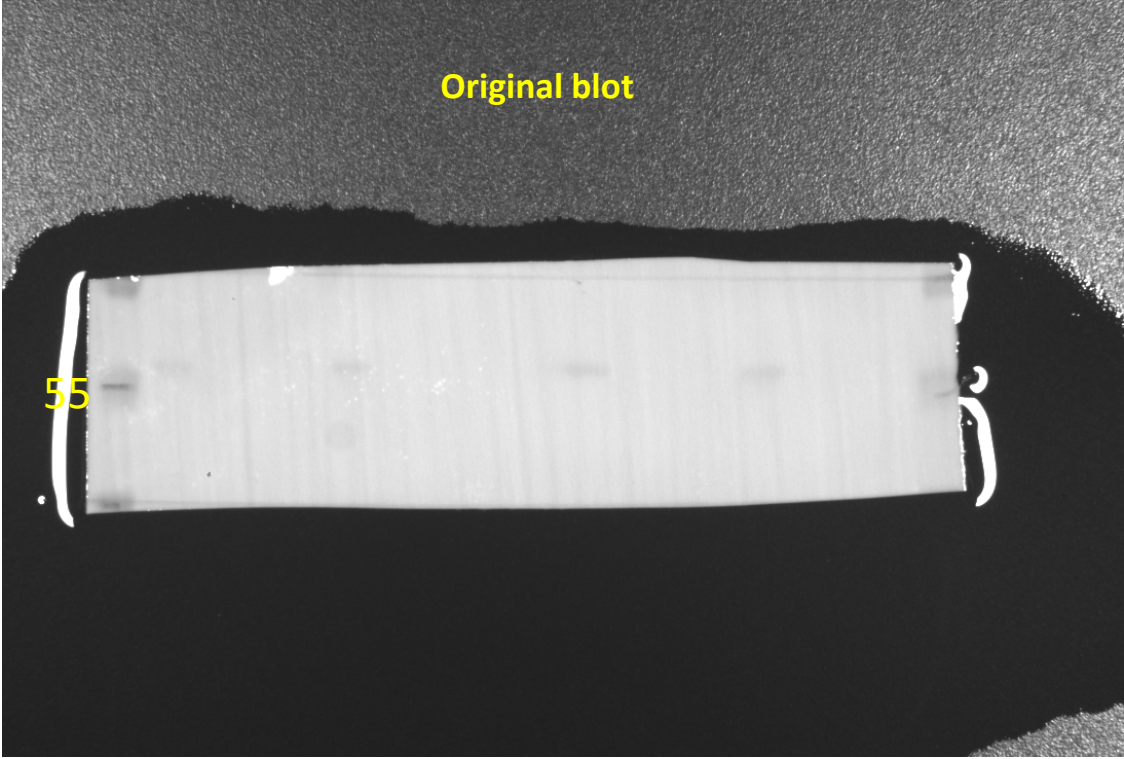

Fig. 5d

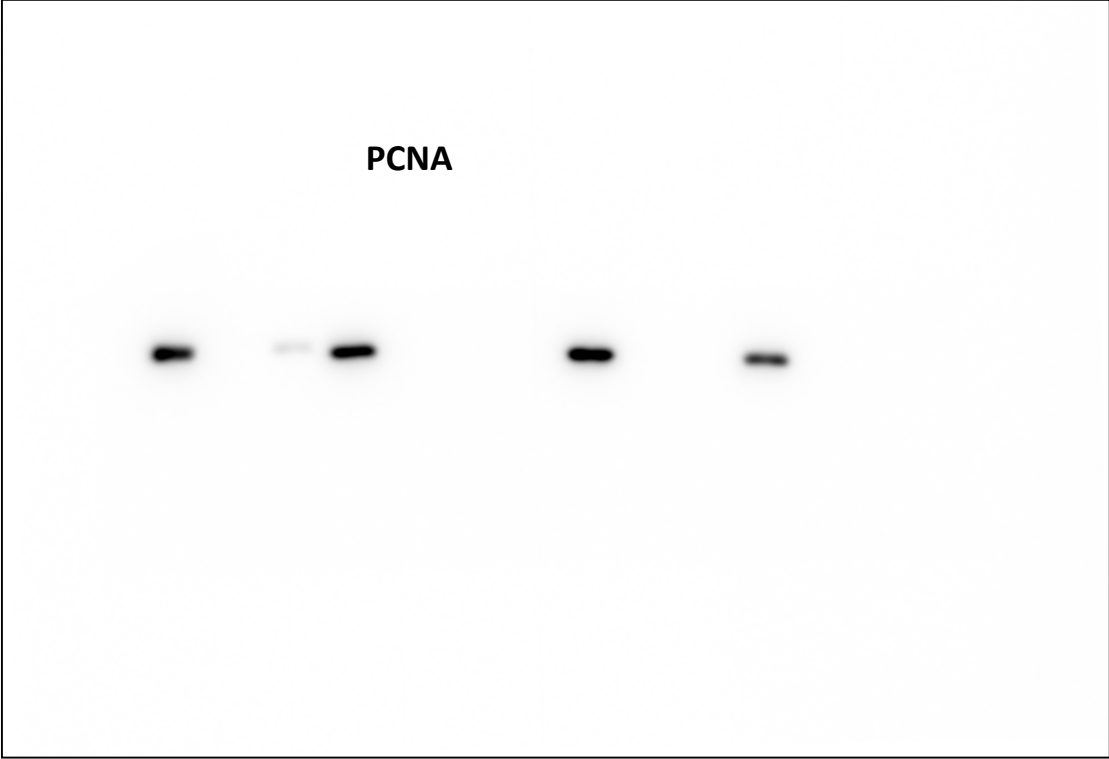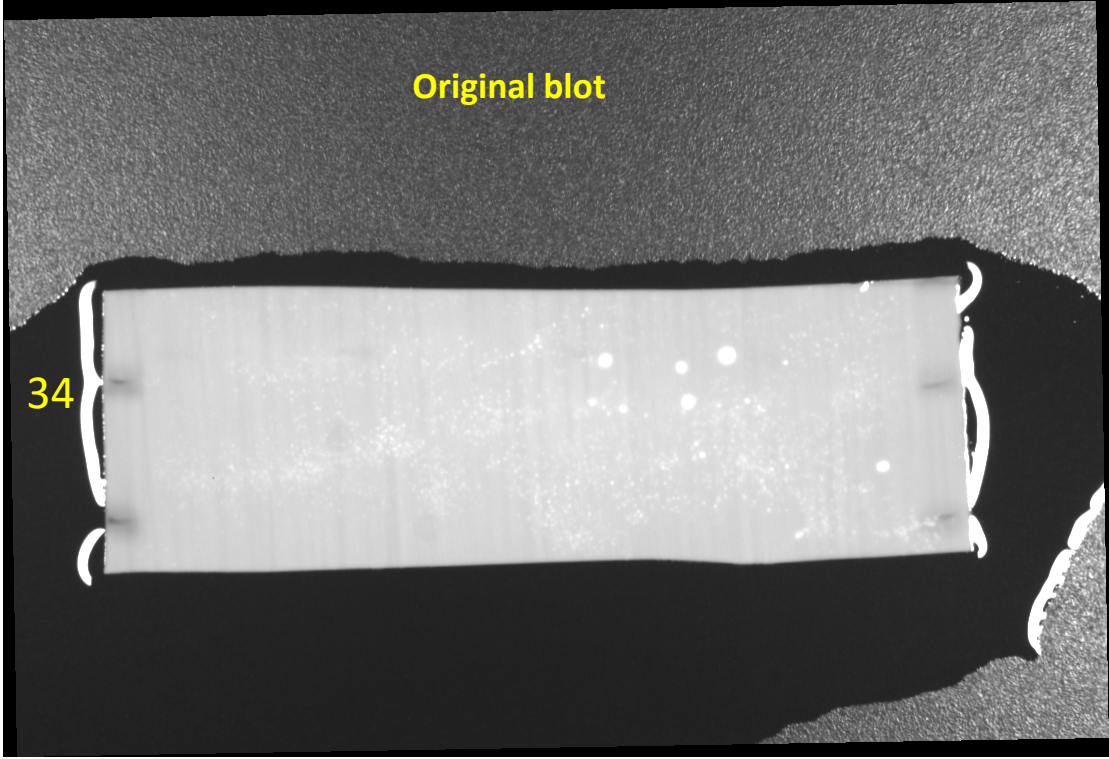

Fig. 6c

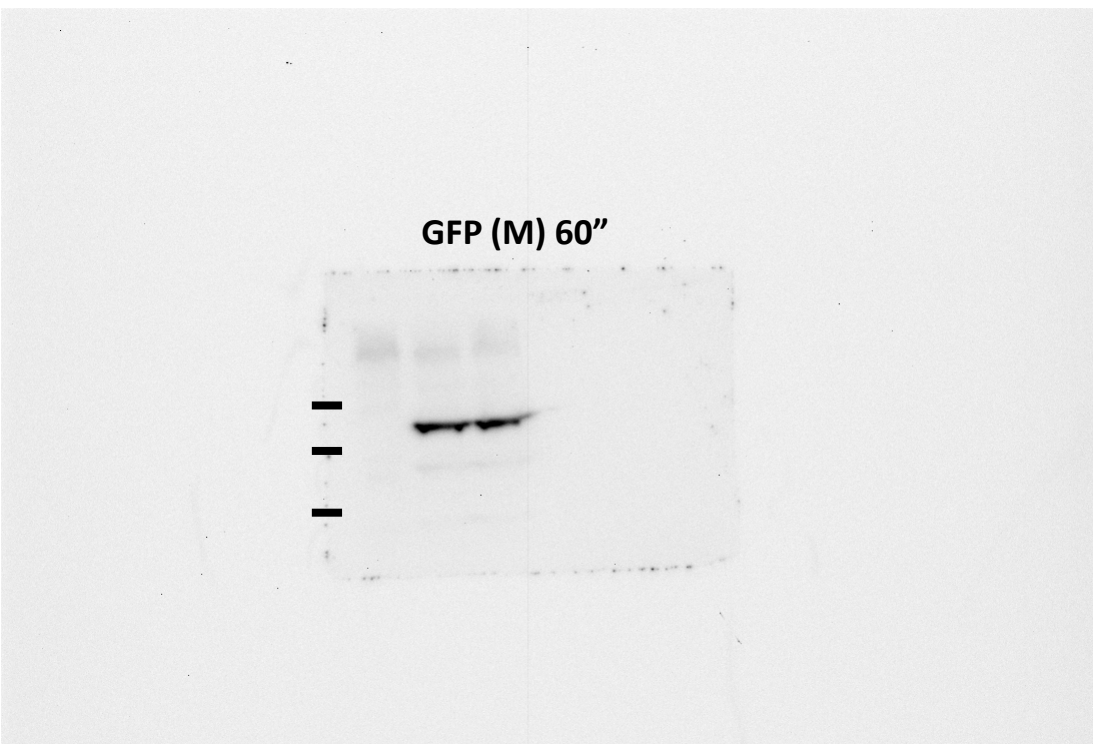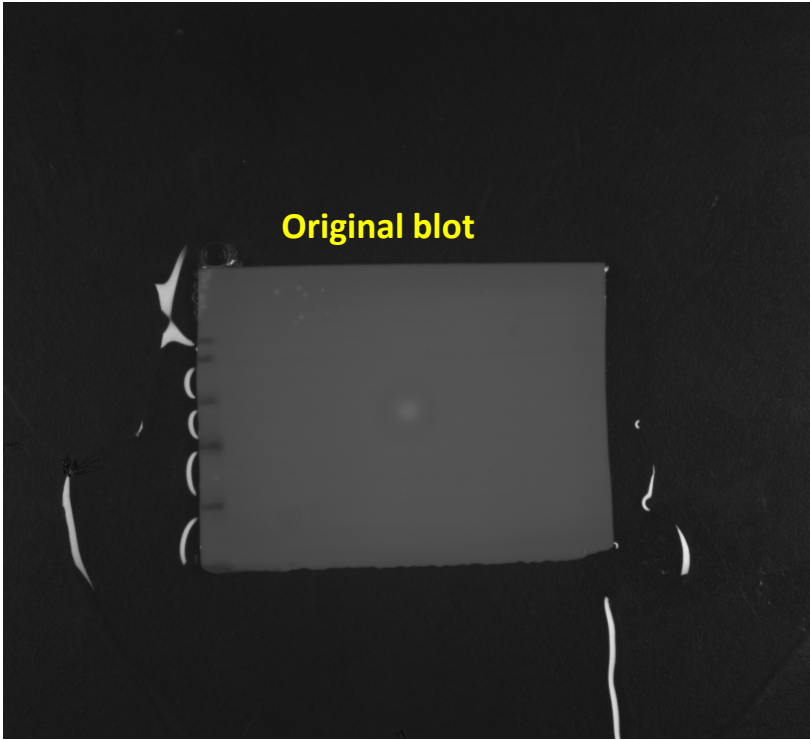

Fig. 6c

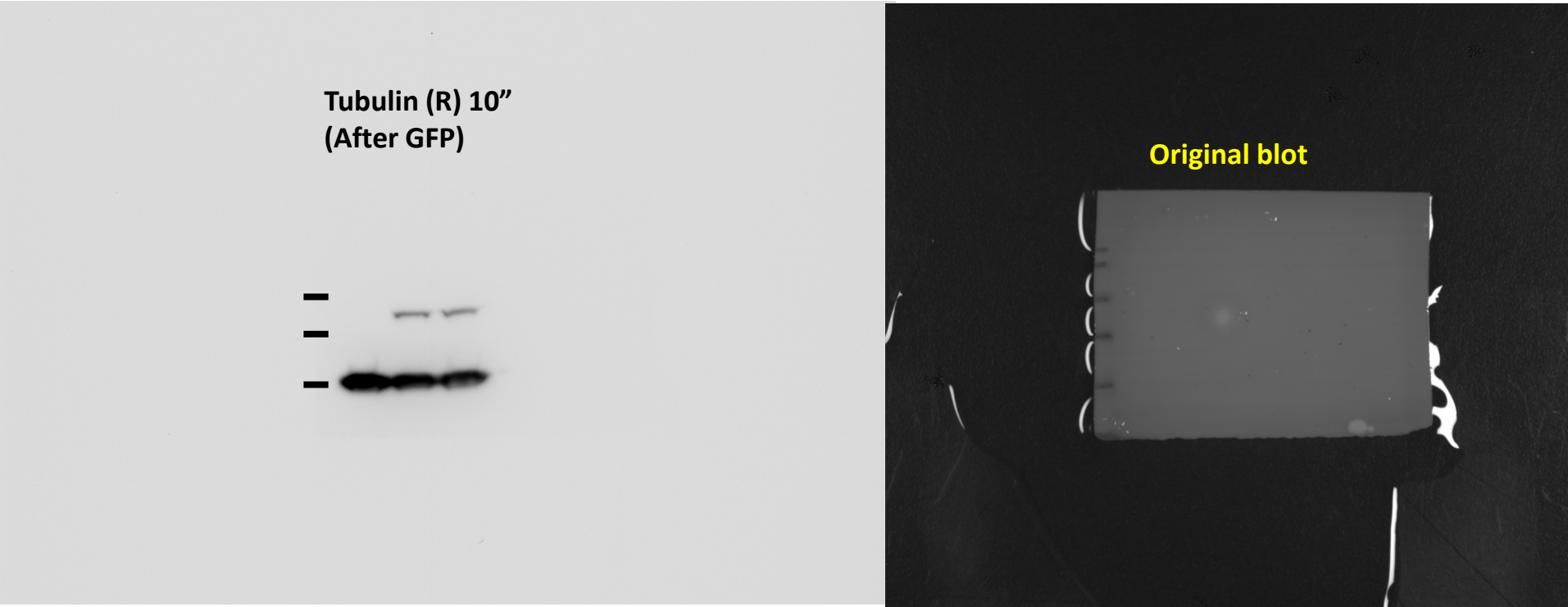

Fig. 6c

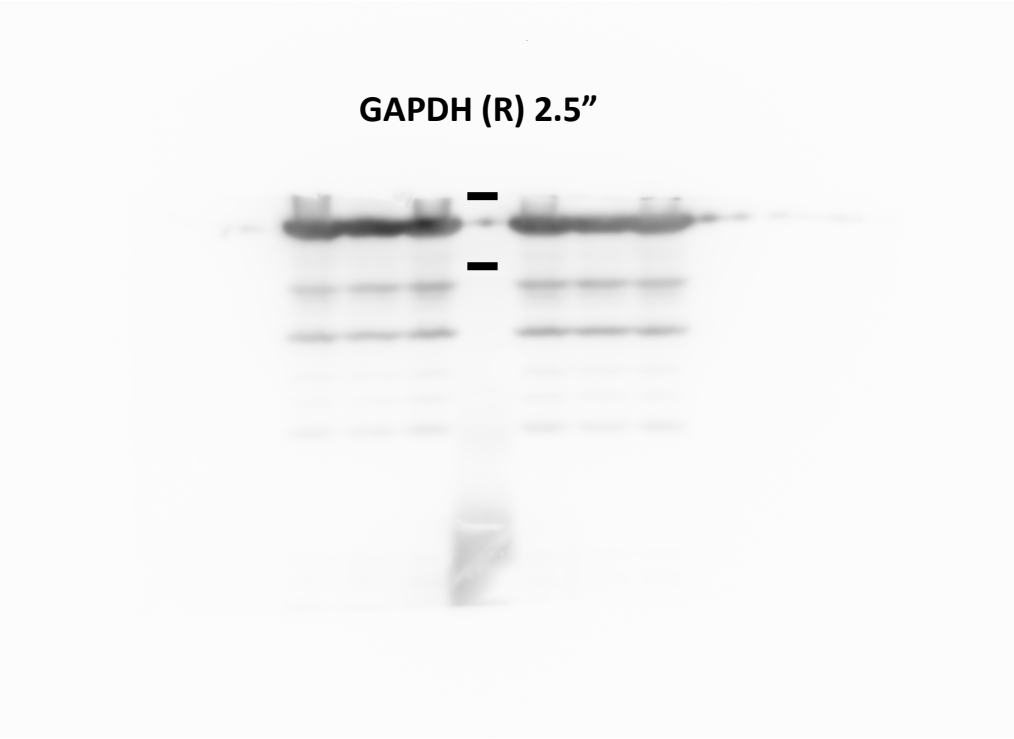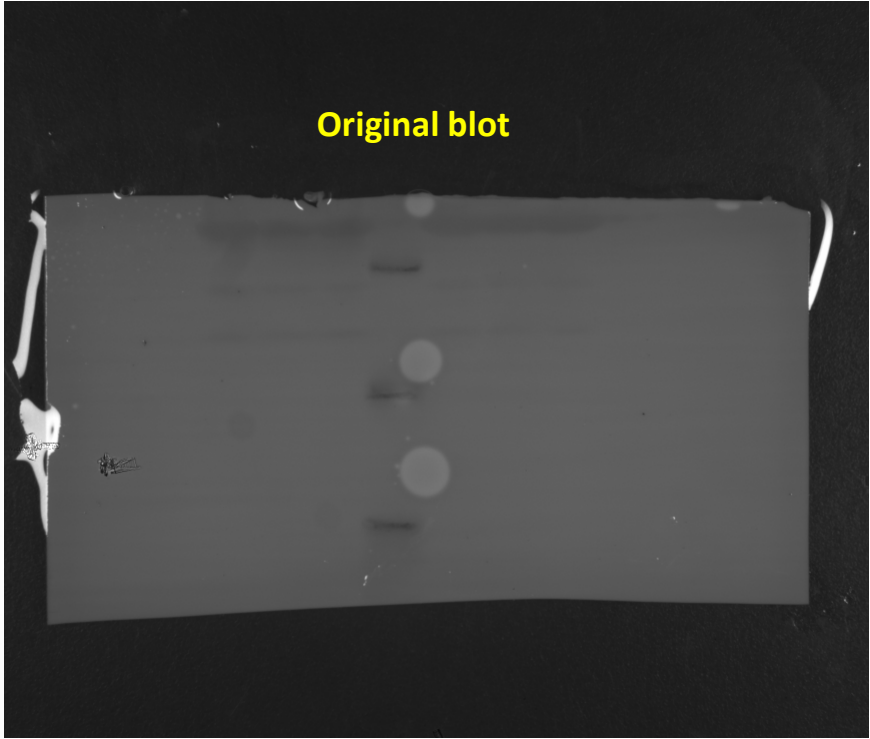

Supplement: Supplementary file 1 — Original blots [file 41420_2023_1301_MOESM1_ESM.pdf]
